# Restriction by SAMHD1 Limits cGAS/STING-Dependent Innate and Adaptive Immune Responses to HIV-1

**DOI:** 10.1016/j.celrep.2016.07.002

**Published:** 2016-07-28

**Authors:** Jonathan Maelfait, Anne Bridgeman, Adel Benlahrech, Chiara Cursi, Jan Rehwinkel

**Affiliations:** 1 Medical Research Council Human Immunology Unit, Weatherall Institute of Molecular Medicine and Radcliffe Department of Medicine, University of Oxford, Oxford OX3 9DS, UK; 2 Medical Research Council Human Immunology Unit, Weatherall Institute of Molecular Medicine and Nuffield Department of Medicine, University of Oxford, Oxford OX3 9DS, UK

## Abstract

SAMHD1 is a restriction factor for HIV-1 infection. *SAMHD1* mutations cause the autoinflammatory Aicardi-Goutières syndrome that is characterized by chronic type I interferon (IFN) secretion. We show that the spontaneous IFN response in SAMHD1-deficient cells and mice requires the cGAS/STING cytosolic DNA-sensing pathway. We provide genetic evidence that cell-autonomous control of lentivirus infection in myeloid cells by SAMHD1 limits virus-induced production of IFNs and the induction of co-stimulatory markers. This program of myeloid cell activation required reverse transcription, cGAS and STING, and signaling through the IFN receptor. Furthermore, SAMHD1 reduced the induction of virus-specific cytotoxic T cells in vivo. Therefore, virus restriction by SAMHD1 limits the magnitude of IFN and T cell responses. This demonstrates a competition between cell-autonomous virus control and subsequent innate and adaptive immune responses, a concept with important implications for the treatment of infection.

## Highlights

• Spontaneous IFN production in SAMHD1-deficient cells requires cGAS and STING
• During HIV-1 infection, SAMHD1 limits activation of myeloid cells
• cGAS and STING detect HIV-1 infection in SAMHD1-deficient cells and induce IFN
• SAMHD1 prevents virus-specific CD8 T cell responses in vivo

## Graphical Abstract

**Figure:**
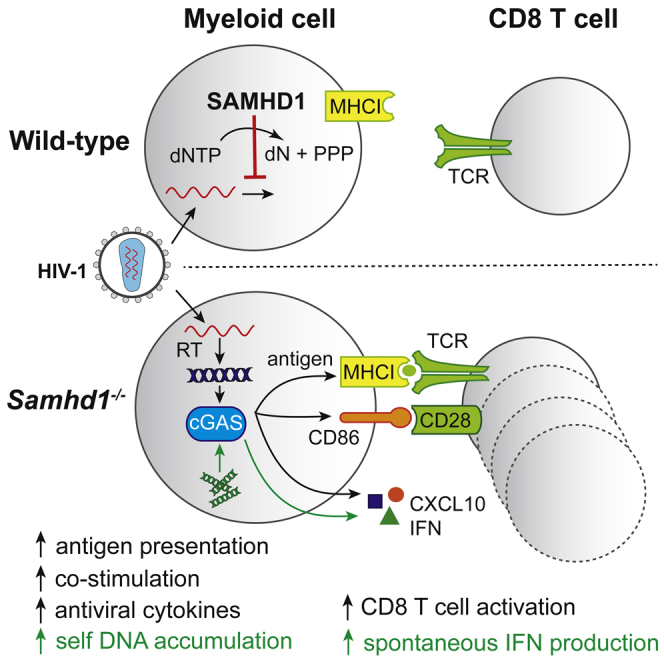

## Teaser

Restriction factors defend infected cells against viruses. Maelfait et al. find that SAMHD1, which blocks HIV-1 infection in myeloid cells, prevents innate sensing of infection via cGAS/STING. Furthermore, SAMHD1 curtails virus-specific T cell responses in vivo. Restriction by SAMHD1, therefore, limits subsequent innate and adaptive immune responses.

## Introduction

Virus infection in mammalian hosts is controlled by a variety of mechanisms operating at different levels. These include cell-intrinsic restriction systems, innate immune sensors that signal for the induction of an antiviral state, and cellular and adaptive immune responses. How these different branches of the antiviral response work together is important for successful immunity. The role of pattern-recognition receptors that sense infection for the development of subsequent immune responses has been well documented (Medzhitov, 2009). However, less is known about how virus control by restriction factors is linked with innate and adaptive immune responses.

Restriction factors have been studied in particular detail for HIV-1 and include APOBEC3G, TRIM5α, tetherin, and Mx2 (Rehwinkel, 2014, Simon et al., 2015). Another HIV-1 restriction factor is SAMHD1, a deoxynucleoside triphosphate (dNTP) triphosphohydrolase that depletes the intracellular pool of dNTPs and thereby prevents HIV-1 reverse transcription in some cell types (Ayinde et al., 2012). Additional mechanisms by which SAMHD1 might restrict infection have been proposed and include degradation and/or binding of viral nucleic acids (Ballana and Esté, 2015).

Several studies suggested that SAMHD1-deficient cells produce elevated levels of type I interferons (IFNs) in response to HIV-1 infection. Indirect evidence for this idea stems from experiments using Vpx, a viral accessory protein encoded by HIV-2, but not HIV-1. Vpx targets SAMHD1 for proteasomal degradation (Hrecka et al., 2011, Laguette et al., 2011). Depletion of SAMHD1 by Vpx in cultured human cells not only facilitates HIV-1 infection but also results in the induction of an antiviral response (Manel et al., 2010). In addition, cells from patients with *SAMHD1* mutations or cells in which SAMHD1 is depleted by RNAi produce more IFNs during HIV-1 infection (Berger et al., 2011, Puigdomènech et al., 2013). Subsequent work identified a role for cytosolic DNA sensing by cGAS and STING in IFN induction in Vpx-treated cells (Gao et al., 2013, Lahaye et al., 2013). Furthermore, SAMHD1 depletion in vitro in human dendritic cells (DCs) by Vpx delivery or RNAi enhances DC activation and antigen presentation upon HIV-1 infection and facilitates T cell responses in co-culture models (Ayinde et al., 2015). However, the interpretation of these data is complicated by the possibility that Vpx targets additional proteins apart from SAMHD1 (Fujita et al., 2012, Reinhard et al., 2014), by genetic heterogeneity of patients’ cells, and by recent results that failed to reproduce enhanced DC activation in HIV-1-infected cells depleted of SAMHD1 (Hertoghs et al., 2015). In vivo data and genetic studies in knockout models interrogating the possible role of SAMHD1 in innate and adaptive immune responses to HIV-1 are currently lacking.

Mutations in human *SAMHD1* cause Aicardi-Goutières syndrome (AGS), a rare monogenic disorder resembling congenital virus infection and typified by early-onset brain disease (Rice et al., 2009). AGS patients spontaneously produce IFNs in the absence of infection with exogenous viruses (Crow and Manel, 2015). These observations suggest that SAMHD1 prevents the accumulation of endogenous nucleic acids that induce IFNs. Others and we previously reported spontaneous IFN production in *Samhd1*^−/−^ mice and cells, mimicking the situation in AGS patients (Behrendt et al., 2013, Rehwinkel et al., 2013). The identity and source of the cellular IFN-stimulatory nucleic acid and the signaling pathway activated by it are important open questions (Roers et al., 2016).

We show that cGAS and STING mediate chronic IFN production in *Samhd1*^−/−^ mice and cells. We further show that SAMHD1-deficient myeloid cells produce heightened levels of IFNs in response to infection with VSV-G-pseudotyped HIV-1-derived lentiviruses and are activated more strongly. These effects required reverse transcription and were nullified in cells from *Samhd1*^−/−^ mice also lacking cGAS, STING, or IFNAR. We also demonstrate that the induction of antigen-specific T cell responses is limited by SAMHD1 in vivo. Therefore, virus control by SAMHD1 at the level of the infected cell limits innate and adaptive immunity.

## Results

### The Spontaneous IFN Response in *Samhd1*^−/−^ Cells and Mice Requires cGAS and STING

AGS is a prototypical interferonopathy, a group of diseases characterized by chronic and pathologic IFN production (Crow and Manel, 2015). Mutation of *SAMHD1* or one of at least six other genes, including *TREX1* and *RNASEH2A-*C, causes AGS (Crow and Manel, 2015). Others and we previously showed that *Samhd1*^−/−^ mice and cells display a spontaneous IFN signature, although SAMHD1-deficient animals do not develop autoimmunity (Behrendt et al., 2013, Rehwinkel et al., 2013). Given that SAMHD1’s enzymatic function is to degrade dNTPs, the building blocks of DNA, we hypothesized that cytosolic DNA sensing might account for IFN induction in the absence of SAMHD1.

**Figure 1:**
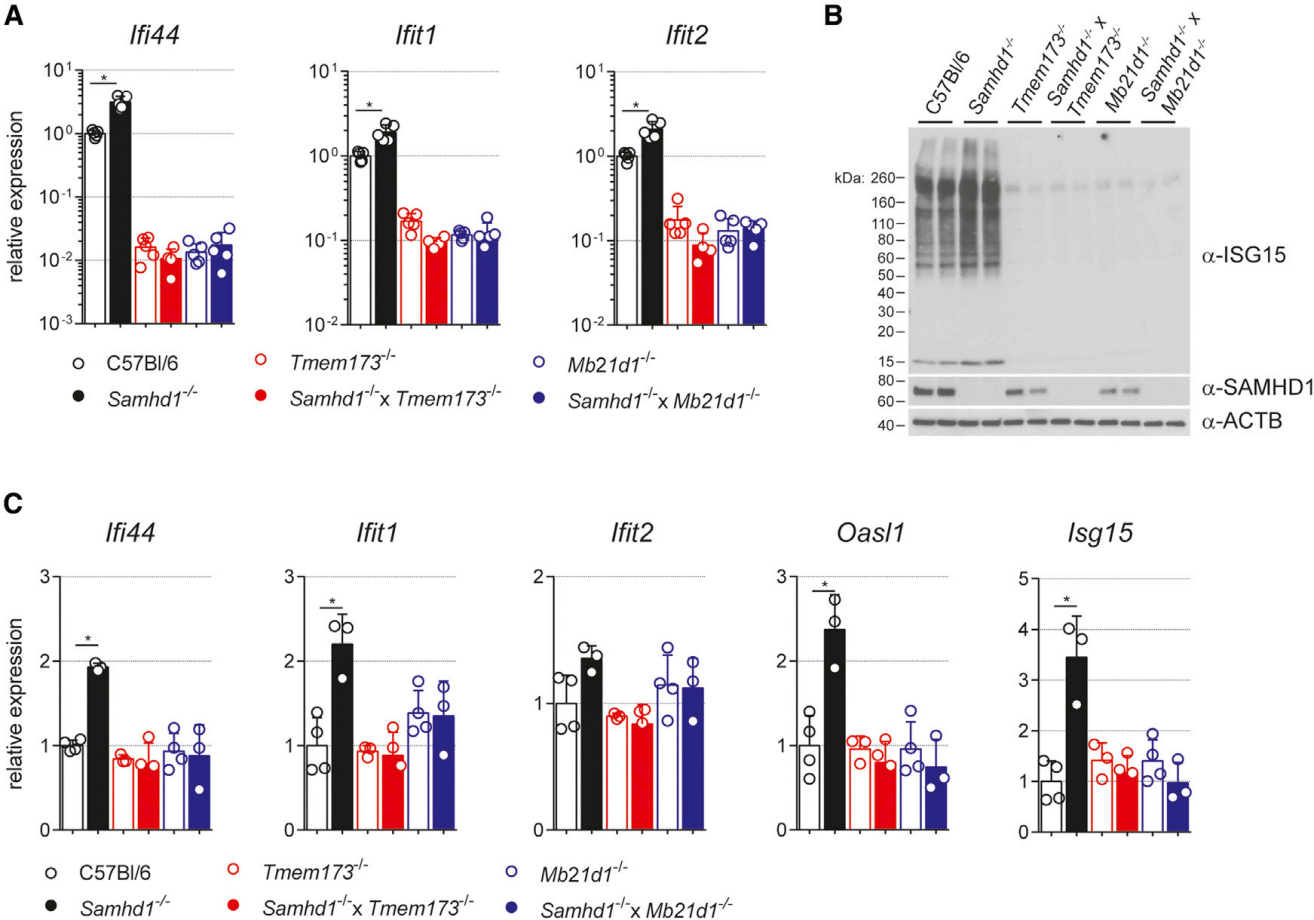
Loss of SAMHD1 Triggers a Spontaneous cGAS/STING-Dependent IFN Response (A and B) BMDMs of the indicated genotypes were cultured for 12 days. (A) mRNA expression of the indicated ISGs by RT-qPCR. Data are presented as fold changes compared to the average of wild-type (C57Bl/6) samples. Each open circle represents mean gene expression from two BMDM cultures from one mouse (n = 5). (B) Western blot for ISG15, SAMHD1, and β-ACTIN (ACTB) using protein lysates from BMDMs. High-molecular-weight signals represent ISGylated proteins. (C) mRNA expression of the indicated ISGs by RT-qPCR. Data are presented as fold changes compared to the mean of wild-type samples. Open circles represent gene expression values from individual 6-month-old mice. At least three mice were analyzed per genotype. Data in (A) and (B) are representative of two independent experiments. Data in (A) and (C) represent mean ± SD (^∗^p < 0.05, Student’s t test).

To test this, we first cultured bone marrow-derived macrophages (BMDMs) from SAMHD1-cGAS (*Mb21d1*^−/−^) or SAMHD1-STING (*Tmem173*^−/−^) double-knockout mice. Consistent with previous observations, *Samhd1*^−/−^ BMDMs expressed higher mRNA levels of the interferon-stimulated genes (ISGs) *Ifi44*, *Ifit1*, and *Ifit2* compared to wild-type cells (Figure 1A). However, the expression of these ISGs was not increased in cells lacking both SAMHD1 and STING or cGAS compared to single-knockout control cells (Figure 1A). It is noteworthy that basal ISG expression was reduced in cGAS- and STING-deficient cells. Wild-type BMDMs thus maintain basal expression of ISGs and this requires an intact cytosolic DNA-sensing pathway.

ISG15 is an IFN-inducible ubiquitin-like protein modifier with antiviral functions (Morales and Lenschow, 2013). *Samhd1*^−/−^ cells expressed more ISG15 protein and contained increased amounts of high-molecular-weight proteins, which were covalently modified by ISG15 (Figure 1B). Enhanced ISGylation was not observed in SAMHD1-deficient BMDMs that did not express cGAS or STING (Figure 1B).

To confirm these findings in vivo, we analyzed spleens from 6-month-old *Samhd1*^−/−^ mice for the expression of several ISGs. The mRNA levels of *Ifi44*, *Ifit1*, *Oasl1*, and *Isg15* were significantly increased in *Samhd1*^−/−^ spleens (Figure 1C). However, the expression of these genes was reduced to wild-type levels when SAMHD1 was knocked out together with cGAS or STING (Figure 1C). In sum, these data show that SAMHD1 prevents spontaneous engagement of the cGAS/STING pathway.

### SAMHD1 Limits IFN Induction and Myeloid Cell Activation upon Lentivirus Infection

Vpx-mediated degradation of SAMHD1 enables productive infection of human DCs and relieves a block to IFN induction and DC activation (Figure S1) (Manel et al., 2010). To test this in a genetically tractable system, we generated bone marrow-derived myeloid cells (BMMCs) from wild-type and *Samhd1*^−/−^ mice (Helft et al., 2015). Others and we previously showed that SAMHD1 restricts infection of these cells with HIV-1-based lentiviruses (Behrendt et al., 2013, Rehwinkel et al., 2013). Profound SAMHD1-dependent restriction both in vitro and in vivo was only observed when the *pol* gene carried a point mutation (V148I), which decreases the affinity of the viral RT for dNTPs (Rehwinkel et al., 2013). Second-generation lentiviruses used in our previous study (SGLenti-RT^V148I^), which have a minimal genome encoding only EGFP (Figure S2A), were efficiently restricted by SAMHD1, but they did not induce BMMC activation or IFN secretion (Figures S2C–S2E) (Rehwinkel et al., 2013).

**Figure 2:**
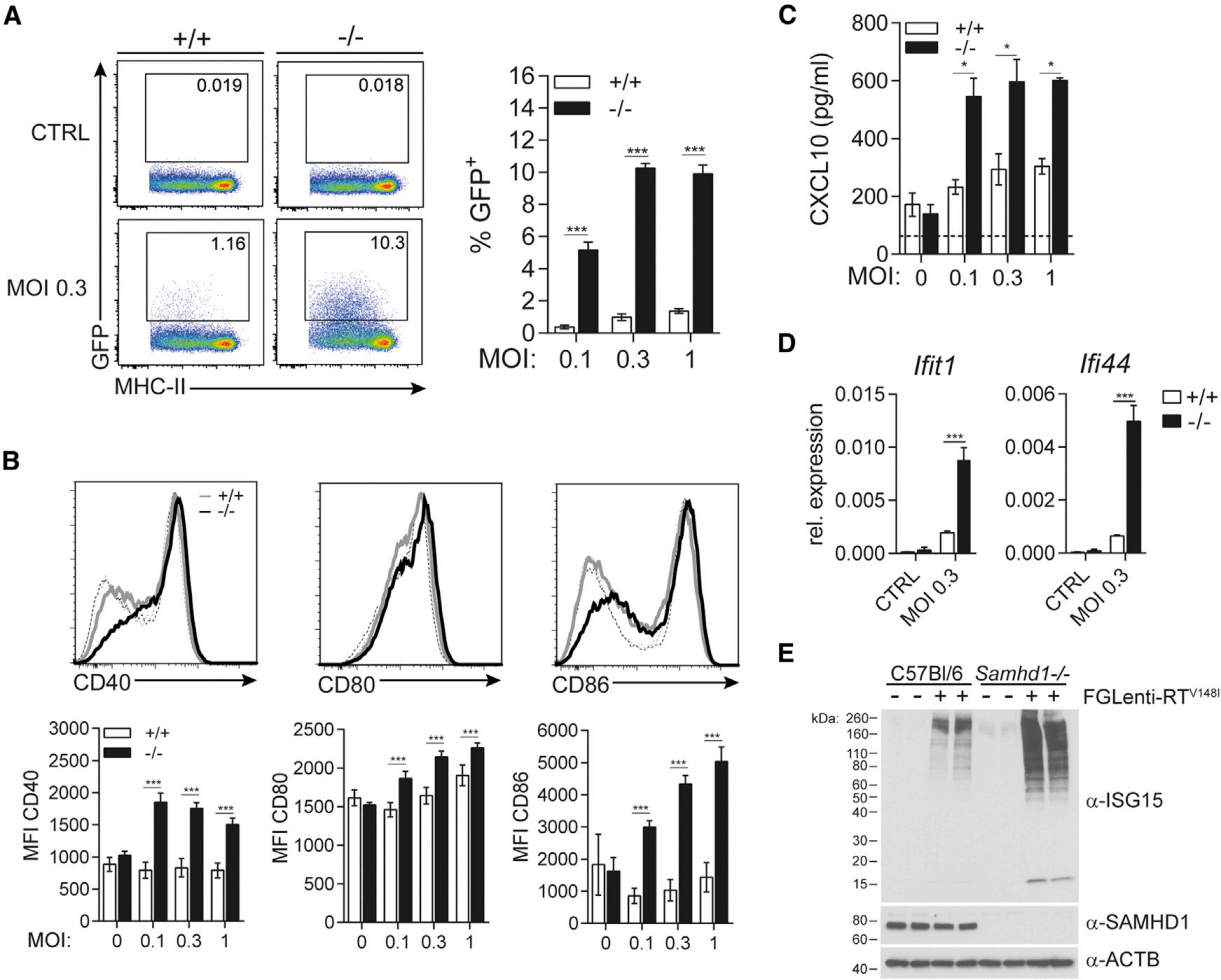
SAMHD1 Limits Infection of BMMCs with First-Generation Lentiviruses and Prevents Innate Sensing (A–E) Wild-type (+/+) or *Samhd1*^−/−^ (−/−) BMMCs were infected for 48 hr with FGlenti-RT^V148I^ using the indicated MOIs. (A) Infection was assessed by flow cytometry. BMMC cultures were gated on CD11c^+^ MHC-II^+^ cells (see Figure S2B). The fraction of GFP^+^ cells was then determined. Representative FACS plots (left) and percentage of GFP^+^ cells are shown (right) (n = 4). (B) Activation of CD11c^+^ MHC-II^+^ BMMCs was analyzed by cell surface staining for CD40, CD80, and CD86. Upper panels depict representative histograms of non-infected (dashed lines) and infected (solid lines, MOI = 0.3) cells. CD40, CD80, and CD86 median fluorescence intensity (MFI) of cells infected with different MOIs is quantified in the bottom graphs (n = 4). (C) CXCL10 in cell culture supernatant was measured by ELISA (n = 4). (D) *Ifit1* and *Ifi44* mRNA expression was analyzed by RT-qPCR in bulk BMMC cultures (n = 4). (E) ISG15, SAMHD1, and β-ACTIN (ACTB) were analyzed by western blot using protein lysates from bulk BMMC cultures (MOI = 0.3). Data are representative of three (A–C) and two (D and E) independent experiments. Data in (A)–(D) represent mean ± SD (^∗^p < 0.05 and ^∗∗∗^p < 0.001, two-way ANOVA). See also Figures S1 and S2.

We therefore infected murine BMMCs with first-generation lentiviruses (FGlenti) that induce IFN in human monocyte-derived dendritic cells (MDDCs) (Figure S1). To allow for potent restriction by SAMHD1, we introduced the V148I point mutation into the *pol* gene of FGLenti (Figure S2A). Infection of BMMCs with both first- and second-generation lentiviruses was equally suppressed by SAMHD1 (Figures 2A and S2C). However, only FGLenti-RT^V148I^ induced cell surface expression of the co-stimulatory molecules CD40, CD80, and CD86 and secretion of IFNα and the chemokine CXCL10, and these effects were significantly elevated in SAMHD1-deficient cells (Figures 2B, 2C, S2D, and S2E). *Ifit1* and *Ifi44* mRNA and ISG15 protein expression and conjugation were enhanced in SAMHD1-deficient BMMCs (Figures 2D and 2E). Taken together, these data provide direct genetic evidence that SAMHD1-mediated restriction impedes detection of lentivirus infection in myeloid cells.

### Viral cDNA Synthesis Is Required for Myeloid Cell Activation

**Figure 3:**
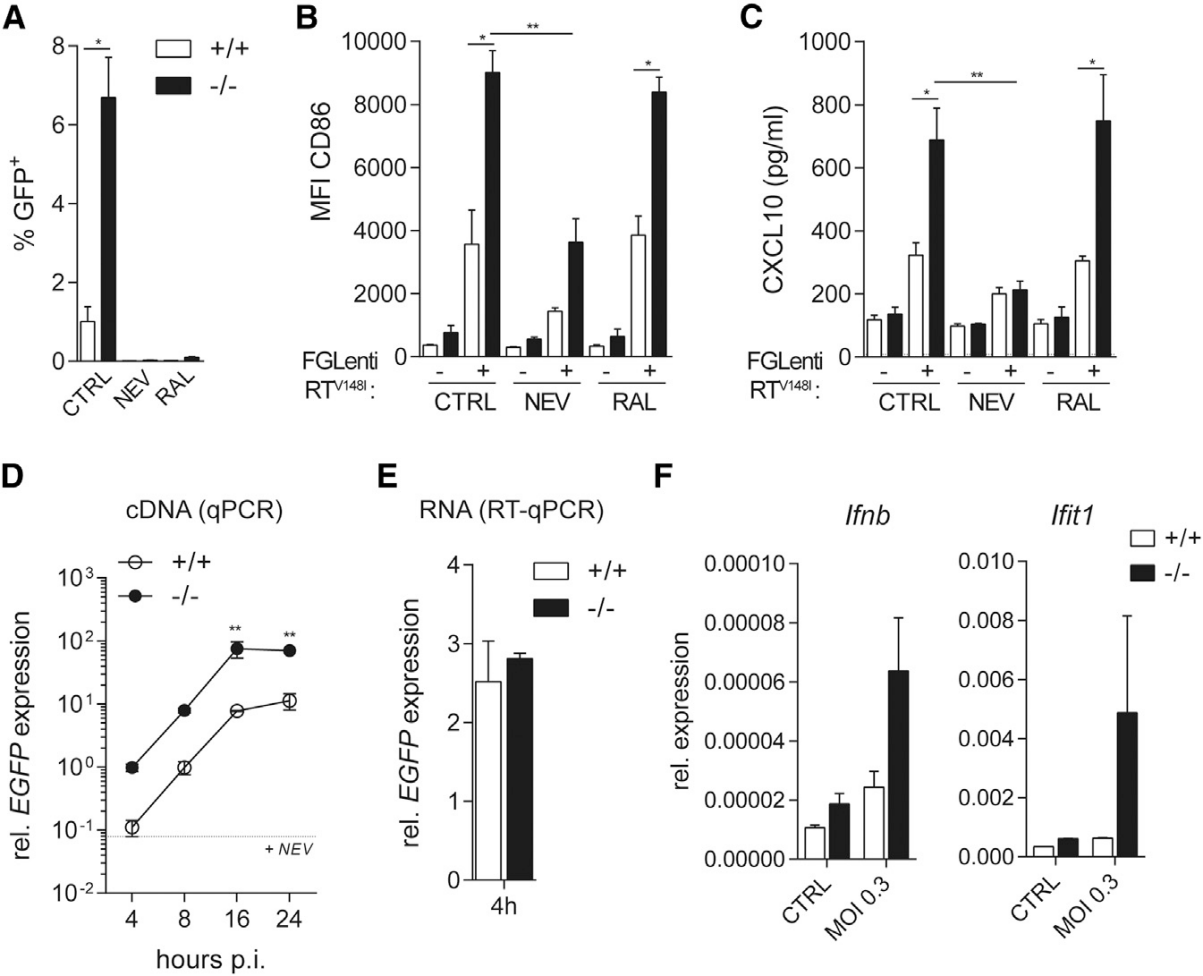
Sensing of First-Generation Lentivirus in *Samhd1*^−/−^ BMMCs Requires the Accumulation of Reverse Transcription Products (A–F) Wild-type (+/+) or *Samhd1*^−/−^ (−/−) BMMCs were infected with FGlenti-RT^V148I^ (MOI = 0.3) in the presence of nevirapine (NEV) or raltegravir (RAL) (A–C). (A) Infectivity was measured as in Figure 2A 48 hr after infection (n = 4). (B) Cell surface expression of CD86 was assessed as in Figure 2B 48 hr after infection (n = 4). (C) CXCL10 in cell culture supernatants was measured by ELISA 48 hr after infection (n = 4). (D) cDNA production was measured by qPCR. Total DNA was extracted from BMMCs at different hours post-infection (p.i.). The dashed line depicts *EGFP* expression in wild-type BMMCs at 16 hr p.i. in the presence of NEV as a control for plasmid contamination of the virus preparation (n = 2). (E) Viral genomic RNA was measured by RT-qPCR using total RNA extracted from BMMCs at 4 hr p.i. (F) *Ifnb* and *Ifit1* mRNA expression was analyzed by RT-qPCR in bulk BMMC cultures at 4 hr p.i. (n = 2). Data are representative of three (A–C) or two (D–F) independent experiments. Data in (A)–(F) represent mean ± SD (^∗^p < 0.05 and ^∗∗^p < 0.01; one-way ANOVA, A–C; two-way ANOVA, D and F; unpaired t test, E). See also Figure S3.

To determine at which step in the viral life cycle HIV-1 is sensed in SAMHD1-deficient myeloid cells, we treated BMMCs with the RT inhibitor nevirapine or the integrase inhibitor raltegravir during infection with FGLenti-RT^V148I^. Both compounds inhibited infection (Figure 3A), but only inhibition of reverse transcription prevented BMMC activation and CXCL10 and IFNα production (Figures 3B, 3C, S3A, and S3B). SAMHD1 may restrict HIV by multiple mechanisms, including by preventing cDNA synthesis and by degrading incoming viral genomic RNA. We found that HIV-1 cDNA accumulated over 24 hr at 10-fold higher levels in *Samhd1*^−/−^ BMMCs compared to wild-type cells (Figure 3D). In contrast, viral RNA levels measured at 4 hr post-infection were not affected by SAMHD1 (Figure 3E). At this time point we observed higher induction of *Ifnb* and *Ifit1* mRNAs in SAMHD1-deficient BMMCs (Figure 3F). These results indicate that viral RNAs do not trigger the IFN response at this time point, which instead depends on reverse transcription and correlates in magnitude with reverse transcription products.

### Lentivirus-Induced Myeloid Cell Activation and IFN Induction Are Dependent on cGAS/STING and IFN Signaling

**Figure 4:**
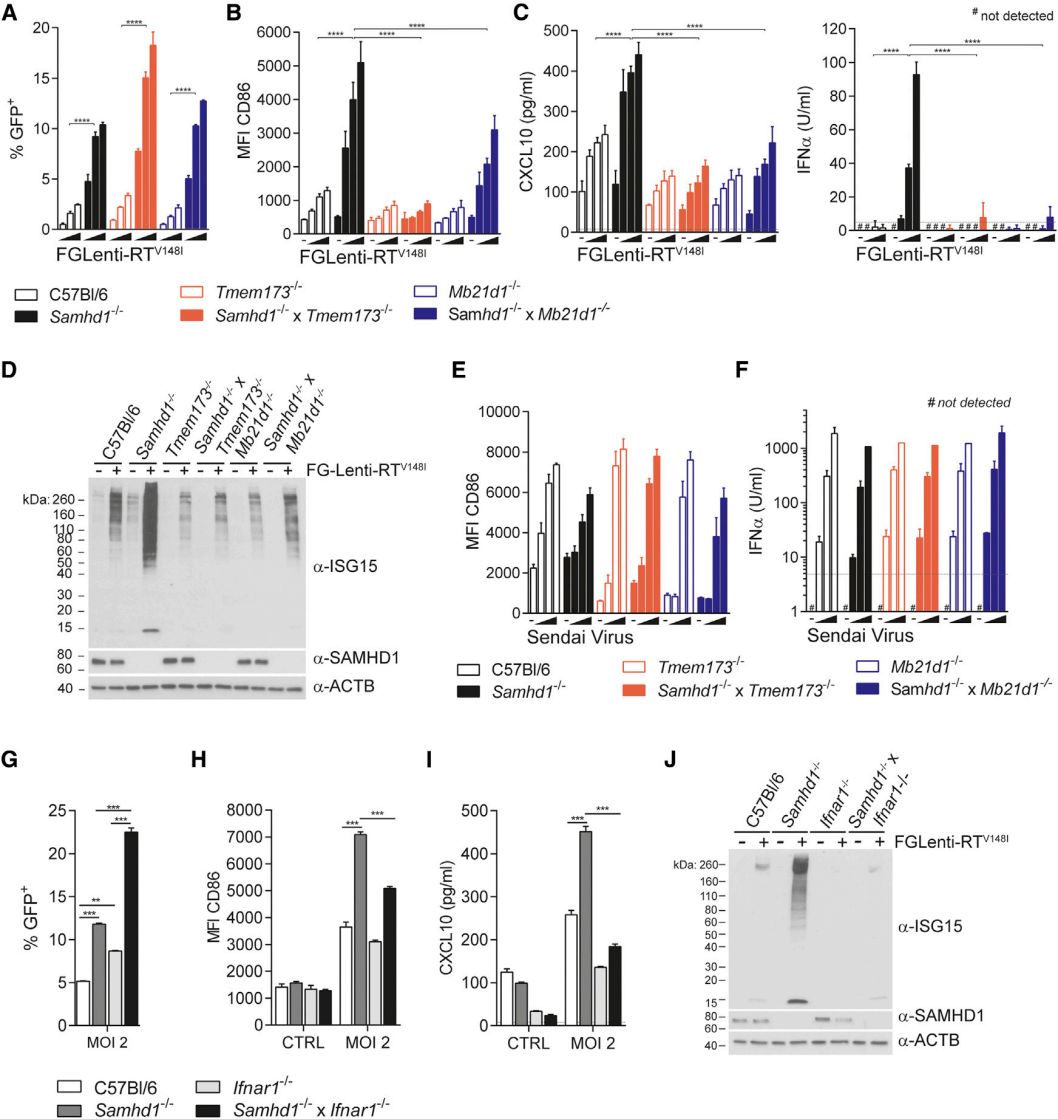
BMMC Activation by First-Generation Lentivirus Is Dependent on cGAS, STING, and IFN Signaling (A–J) BMMCs of the indicated genotypes were infected for 48 hr with FGLenti-RT^V148I^ or SeV. Wedges in (A)–(C), (E), and (F) represent increasing MOIs of FGLenti-RT^V148I^ (0.1, 0.3, and 1) or SeV (0.02, 0.1, and 0.5). *Tmem173*, *Mb21d1*, and *Ifnar1* are the genes encoding STING, cGAS, and type I IFN receptor. (A) Infectivity was measured as in Figure 2A (n = 4). (B) Cell surface expression of CD86 was assessed as in Figure 2B (n = 4). (C) CXCL10 and IFNα in cell culture supernatants were measured by ELISA (n = 4). (D) ISG15, SAMHD1, and β-ACTIN (ACTB) were analyzed by western blot (MOI = 0.3). (E) Cell surface expression of CD86 was assessed as in Figure 2B (n = 2). (F) IFNα in cell culture supernatants was measured by ELISA (n = 2). (G) Infectivity was measured as in Figure 2A (n = 2). (H) Cell surface expression of CD86 was assessed as in Figure 2B (n = 2). (I) CXCL10 in cell culture supernatants was measured by ELISA (n = 2). (J) ISG15, SAMHD1, and β-ACTIN (ACTB) were analyzed by western blot. Data are representative of three independent experiments. Data in (A)–(C) and (E)–(I) represent mean ± SD (^∗∗^p < 0.01, ^∗∗∗^p < 0.001, and ^∗∗∗∗^p < 0.0001; two-way ANOVA, A–C, E, F, H, and I; one-way ANOVA, G). See also Figure S4.

Given the requirement for reverse transcription, we tested whether the cytosolic DNA-sensing pathway detects lentivirus infection in SAMHD1-deficient myeloid cells. SAMHD1-cGAS and SAMHD1-STING double-knockout BMMCs were equally or slightly better infected by FGLenti-RT^V148I^ than *Samhd1*^−/−^ cells (Figure 4A). BMMC activation, the secretion of CXCL10 and IFNα, and ISG15 protein expression and conjugation were all greatly reduced in *Samhd1*^−/−^ BMMCs lacking cGAS or STING (Figures 4B–4D and S4A). Neither BMMC activation nor IFNα production were affected by loss of SAMHD1, cGAS, or STING upon infection with Sendai virus (SeV), which is sensed via the RIG-I pathway (Kato et al., 2006) (Figures 4E, 4F, and S4B). Next, we assessed the role of IFN signaling in activation of SAMHD1-deficient myeloid cells by infecting SAMHD1-type I IFN receptor (*Ifnar1*^−/−^) double-knockout BMMCs with FGlenti-RT^V148I^. Despite the fact that loss of the type I IFN receptor further increased susceptibility to FGlenti-RT^V148I^ compared to SAMHD1 single-knockout cells (Figure 4G), BMMC activation and antiviral responses in these cells were impaired (Figures 4H–4J, S4C, and S4D). In sum, these observations provide genetic evidence that cGAS, STING, and signaling through the type I IFN receptor are required for SAMHD1-dependent myeloid cell activation upon lentivirus infection.

### SAMHD1 Reduces the Antiviral CD8 T Cell Response In Vivo

**Figure 5:**
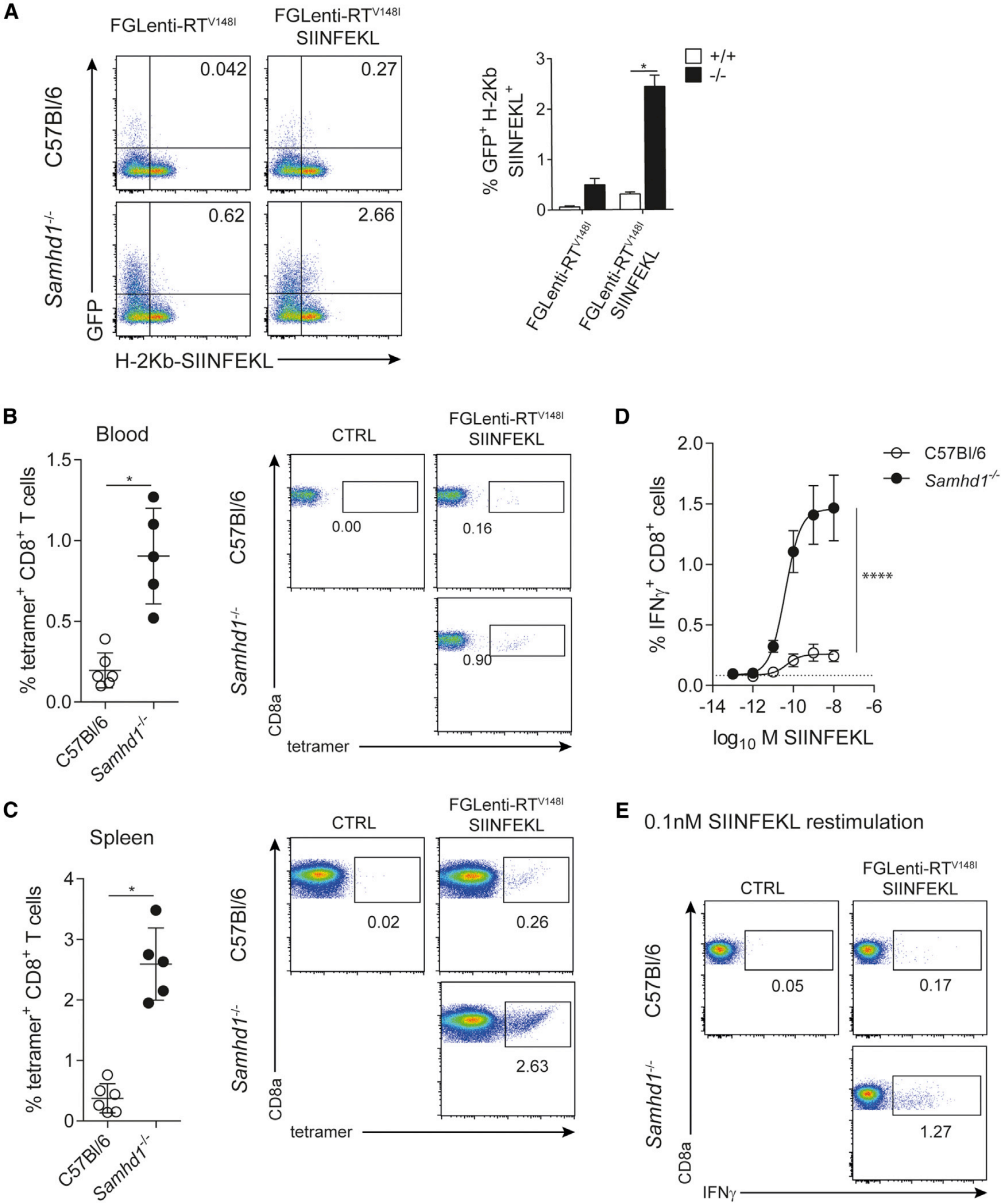
SAMHD1 Prevents Lentivirus-Specific CD8 T Cell Responses In Vivo (A) GFP expression (infectivity) and antigen presentation (using a H2-Kb SIINFEKL-specific antibody) were analyzed by flow cytometry in CD11c^+^ MHC-II^+^ BMMCs 48 hr after infection with FGlenti-RT^V148I^ or FGLenti-RT^V148I^ SIINFEKL (MOI = 0.3). Representative FACS plots are shown on the left and a quantification of infected cells presenting SIINFEKL on MHC-I (GFP^+^ H2-Kb SIINFEKL^+^) is shown in the right panel (n = 4). (B–E) Wild-type (C57Bl/6, n = 6) and *Samhd1*^−/−^ mice (n = 5) were intravenously infected with FGLenti-RT^V148I^ SIINFEKL (100,000 IU/mouse). (B and C) Quantification of H2-Kb-SIINFEKL tetramer^+^ CD8 T cells in the blood 9 days p.i. (B) and in the spleen 10 days p.i. (C). Each dot represents an individual mouse. Representative FACS plots are shown on the right. Numbers indicate percentages of tetramer^+^ CD8 T cells. (D) Intracellular IFNγ staining of spleen cells from (C) after 6 hr of SIINFEKL peptide (10^−13^–10^−8^ M) re-stimulation. Percentages of IFNγ^+^ CD8 T cells are shown. The dashed line represents the fraction of IFNγ^+^ CD8 T cells responding to peptide in an uninfected mouse. (E) Representative FACS plots after re-stimulation with 10^−10^ M SIINFEKL. Numbers indicate percentages of IFNγ^+^ CD8 T cells. Data are representative of two independent experiments. Data in (A)–(D) represent mean ± SD (^∗^p < 0.05 and ^∗∗∗∗^p < 0.0001; unpaired t test, A–C; non-linear 4PL sigmoidal curve fitting, D). See also Figure S5.

Given that SAMHD1 curtails the induction of IFN and the activation of myeloid cells in vitro, we tested whether SAMHD1 limits adaptive immune responses. We fused the OVA_257–264_ peptide SIINFEKL, a model antigen for CD8 T cells (Carbone and Bevan, 1989), to the C terminus of the matrix protein (Figure S5A). The resulting FGLenti-RT^V148I^ SIINFEKL virus behaved identically to the parental virus in terms of virus production, gag processing, infectivity, and BMMC activation (Figures S5B–S5F). Furthermore, SIINFEKL presentation by H-2Kb was enhanced in *Samhd1*^−/−^ cells (Figure 5A).

Next we compared the antigen-specific CD8 T cell response in wild-type and *Samhd1*^−/−^ animals. Compared to wild-type mice, infection of *Samhd1*^−/−^ animals resulted in 4- and 10-fold increased tetramer-positive CD8 T cell populations in blood and spleens, respectively (Figures 5B, 5C, and S5G). Spleens of *Samhd1*^−/−^ mice contained more CD8 T cells producing IFNγ upon SIINFEKL re-stimulation than those of wild-type controls (Figures 5D and 5E), but not after phorbol 12-myristate 13-acetate (PMA) and ionomycin stimulation (Figure S5H). SAMHD1-cGAS double-knockout mice mounted a similar antigen-specific CD8 T cell response compared to mice lacking only SAMHD1 (Figure S5I). This indicates that the increased antigen load in *Samhd1*^−/−^ animals determines the magnitude of the CD8 T cell response in this model. To exclude direct effects of SAMHD1 on the CD8 T cell repertoire or T cell activation, we injected *Samhd1*^−/−^ and wild-type mice with OVA protein using cGAMP as an adjuvant (Li et al., 2013). This resulted in comparable SIINFEKL-specific CD8 T cell responses in wild-type and *Samhd1*^−/−^ mice (Figures S5J and S5K). We conclude that SAMHD1 limits lentivirus-specific T cell responses to lentiviral infection in vivo.

## Discussion

Our study provides genetic and in vivo data that the restriction factor SAMHD1 does not operate in conjunction with other innate and adaptive immune responses to ensure control of lentivirus infection. Instead, SAMHD1 limits the induction of IFNs, ISGs and co-stimulatory molecules as well as the amounts of antigen presented by infected myeloid cells in vitro. Furthermore, SAMHD1 limits the magnitude of the CD8 T cell response induced in vivo. Cell-autonomous virus restriction by SAMHD1, therefore, competes with sensors of infection and T cell responses. This antagonistic relationship between SAMHD1 and innate and adaptive immune responses is of importance for our understanding of lentiviral pathogenicity, and it may provide therapeutic opportunities to control viral infections.

It is interesting to ask whether this concept also applies to other restriction factors. Indeed, a recent study showed that cultured DCs inactivate TRIM5α’s function by redirecting the protein to the cell nucleus, and it suggested that this reflects an evolutionary trade-off in DCs, in which restriction is minimized to allow for more efficient sensing (Portilho et al., 2016). However, other restriction factors do not compete with but rather facilitate subsequent innate and adaptive immune responses. APOBEC3 is required for efficient production of antibodies in retrovirus-infected mice (Santiago et al., 2008, Santiago et al., 2010, Smith et al., 2011), and human APOBEC3G facilitates T cell responses (Casartelli et al., 2010). Tetherin promotes NK and CD8 T cell responses in a mouse model of retrovirus infection (Li et al., 2014). Interestingly, some restriction factors, including TRIM5α and tetherin, also operate as sensors of infection and induce NF-κB (Galão et al., 2012, Pertel et al., 2011). Viral restriction factors thus differentially impact the host’s immune responses, and it is likely that this complexity is due to host-pathogen co-evolution.

A first hint at the possibility that SAMHD1 prevents sensing of virus infection came from its implication in AGS, a rare autoinflammatory disease characterized by chronic IFN production (Rice et al., 2009). Here we show that the spontaneous IFN response in S*amhd1*^−/−^ cells and mice requires cGAS and STING. Together with our previous observation that S*amhd1*^−/−^ cells respond normally to stimulation with exogenous DNA or STING agonists (Rehwinkel et al., 2013), these data suggest that an unusual endogenous DNA accumulates in SAMHD1-deficient cells and engages cGAS. This mirrors the situation in TREX1- and RNASEH2-deficient cells (Roers et al., 2016).

We further report that IFN production in lentivirus-infected *Samhd1*^−/−^ myeloid cells requires cGAS and STING. Induction of antiviral responses was dependent upon reverse transcription, implicating viral reverse-transcribed DNA as the cGAS trigger. These data provide genetic evidence that lentivirus-induced innate immune responses are dependent on cytosolic DNA sensing. As reported for human DCs (Manel et al., 2010), we find that first-generation lentiviruses encoding a full genome and second-generation viruses with a minimal genome differ in their abilities to induce IFN. It has been suggested that newly expressed gag unmasks non-integrated cDNA in the DC cytosol, making it available for detection by cGAS (Lahaye et al., 2013, Manel et al., 2010). Indeed, gag is expressed by first-generation lentiviruses, but not by second-generation viruses. Specific nucleic acid sequences or secondary structures only present in the full HIV-1 genome may also, perhaps in conjunction with additional host proteins, contribute to IFN induction (Herzner et al., 2015, Yoh et al., 2015).

To study the impact of lentivirus restriction by SAMHD1 on cytotoxic T cell responses in an in vivo model, we generated a lentivirus expressing the OVA peptide SIINFEKL. We found that infected *Samhd1*^−/−^ myeloid cells better presented this model antigen. Enhanced antigen presentation also had been observed in HIV-1-infected human DCs upon SAMHD1 depletion with Vpx or RNAi, and these treatments resulted in stronger T cell responses in co-culture models (Ayinde et al., 2015, Manel et al., 2010). These in vitro studies, however, mostly relied on HIV-1-specific memory T cells, which have altered requirements for activation (Berard and Tough, 2002). It was, therefore, important to establish an in vivo model in which naive T cells are primed. Notably, we found that antigen-specific CD8 T cell responses were up to 10-fold stronger in *Samhd1*^−/−^ mice compared to wild-type controls upon infection with the SIINFEKL-encoding lentivirus. It is interesting to ask whether this is due to enhanced antigen levels and/or to augmented co-stimulation and secretion of cytokines by antigen-presenting cells. We found that the SIINFEKL-specific T cell response was similarly enhanced in infected *Samhd1*^−/−^ and *Samhd1*^−/−^; *Mb21d1*^−/−^ animals. Thus, in this setting, increased antigen levels are largely responsible for the effect of SAMHD1 on the T cell response. However, H-2Kb-bound SIINFEKL elicits strong T cell responses due to its exceptionally high affinity for the T cell receptor, and little signals 2 and 3 (co-stimulation and cytokines) are required for the SIINFEKL response (Denton et al., 2011). It will therefore be interesting to test other antigens in the future.

An interesting comparison can be made with HIV-2, which overcomes SAMHD1-mediated restriction by encoding the antagonist Vpx (Hrecka et al., 2011, Laguette et al., 2011). In line with our conclusion that SAMHD1 limits immune responses, in vitro infection of DCs with HIV-2 results in potent IFN induction (Lahaye et al., 2013). In humans, HIV-2 infection in comparison with HIV-1 infection is characterized by better immune control, and fewer HIV-2-infected individuals progress to disease (Nyamweya et al., 2013, Rowland-Jones and Whittle, 2007). It is possible that these effects are due in part to relief of SAMHD1-mediated restriction in HIV-2-infected cells, allowing for better innate and adaptive immune responses, and that HIV-1 has become the more successful pathogen by allowing itself to be restricted by SAMHD1. Consistent with this notion are observations in HIV-1 elite controllers. The expression level of SAMHD1 is reduced in DCs from elite controllers; these cells mount enhanced IFN responses to infection and are superior in vitro in stimulating CD8 T cells (Martin-Gayo et al., 2015).

These observations, together with the genetic evidence presented here for an inhibitory role of SAMHD1 on immune responses, suggest a strategy for the development of an efficient HIV-1 vaccine in the form of a replication-deficient virus that circumvents restriction by SAMHD1. These could be achieved either by inclusion of Vpx or by the development of small molecule inhibitors of SAMHD1. Pharmacological inhibition of SAMHD1 also could be useful in therapeutic approaches aimed at eradicating latent HIV-1. These strategies often rely on re-activation of latent provirus and subsequent killing of the virus by the immune system, which we predict would be more effective in the absence of SAMHD1 (Margolis and Hazuda, 2013). In sum, our work sheds light on the in vivo interplay among different branches of the antiviral immune system, and these insights have important implications for controlling infection.

## Experimental Procedures

### Mice

All mice were on the C57Bl/6 background. *Samhd1*^−/−^ mice were described previously (Rehwinkel et al., 2013). *Ifnar1*^−/−^ mice were a gift from C. Reis e Sousa and were originally described in (Müller et al., 1994). *Tmem173*^−/−^ (STING-deficient) mice were a gift from J. Cambier (Jin et al., 2011). *Mb21d1*^−/−^ (cGAS knockout first) mice were described in Bridgeman et al. (2015). This work was performed in accordance with the UK Animals (Scientific Procedures) Act 1986 and institutional guidelines for animal care. This work was approved by a project license granted by the UK Home Office (PPL No. 40/3583) and also was approved by the Institutional Animal Ethics Committee Review Board at the University of Oxford.

### Virus Production, Infection, and OVA-cGAMP Immunizations

All lentiviruses were produced in TLA HEK293T cells. Cells were transfected using Fugene6 (Promega) using the plasmid combinations listed in Table S2. All viruses were VSV-G pseudotyped. Medium was replaced the day after transfection, and 48 hr later supernatant was collected and filtered through a 0.45-μm polyethersulfone (PES) filter. Virus was concentrated by centrifugation over a 20% sucrose cushion at 50,000 × *g* for 2 hr. Viral titers were determined on TLA HEK293T cells by fluorescence-activated cell sorting (FACS) based on GFP expression. Target cells were infected in the presence of 8 μg/ml polybrene (Sigma-Aldrich). After 2 hr, the inoculum was washed away and replaced with fresh medium. For measuring viral cDNA and RNA, virus was incubated with 10 U/ml DNase I (Turbo DNase I, Thermo Fisher Scientific) for 30 min at 37°C in 1× reaction buffer diluted in RPMI. Then 10 μM nevirapine or raltegravir was added to the cells 30 min prior to infection. Mice were infected by intravenous injection of 100,000 IU of FGLenti-RT^V148I^ SIINFEKL (see Figure S5G for virus titration). For immunization with OVA and cGAMP, mice were anesthetized with isoflurane and injected intramuscularly with 10 μg 2′3′-cGAMP (Biolog, C 161) and 10 μg endotoxin-free OVA (Hyglos, 321000).

### In Vitro Peptide Restimulation Assay

Spleen cells (2 × 10^6^) were stimulated with SIINFEKL peptide (InvivoGen, 10^−13^–10^−8^ M). After 1 hr, 10 μg/ml brefeldin A (Sigma-Aldrich) was added to the cells, and 5 hr later, cells were collected for intracellular IFNγ staining. Cells were stained for cell surface markers and intracellular molecules, and they were fixed and permeabilized in Cytofix/Cytoperm (BD Biosciences) according to the manufacturer’s instructions.

### Statistics

Statistical analysis was performed in GraphPad Prism v7.00 as detailed in the figure legends.

## Author Contributions

J.M. and J.R. conceived the study, designed experiments, analyzed data, and wrote the manuscript. J.M. performed all experiments. A. Bridgeman, A. Benlahrech, and C.C. provided technical help and advice. All authors read and approved the final manuscript.

## Supplemental Information

Document S1. Supplemental Experimental Procedures, Figures S1–S5, and Tables S1–S5

Document S2. Article plus Supplemental Information
