# Supplementary material for: Restriction by SAMHD1 Limits cGAS/STING-Dependent Innate and Adaptive Immune Responses to HIV-1
Source: Cell Rep. 2016 Jul 28;16(6):1492–501. doi: 10.1016/j.celrep.2016.07.002 (PMC4978700; doi:10.1016/j.celrep.2016.07.002)
Supplement: Document S1. Supplemental Experimental Procedures, Figures S1–S5, and Tables S1–S5 [file mmc1.pdf]

Cell Reports, Volume *16*

Supplemental Information

Restriction by SAMHD1 Limits cGAS/STING-Dependent Innate and Adaptive Immune Responses to HIV-1

Jonathan Maelfait, Anne Bridgeman, Adel Benlahrech, Chiara Cursi, and Jan Rehwinkel

Supplemental Figures

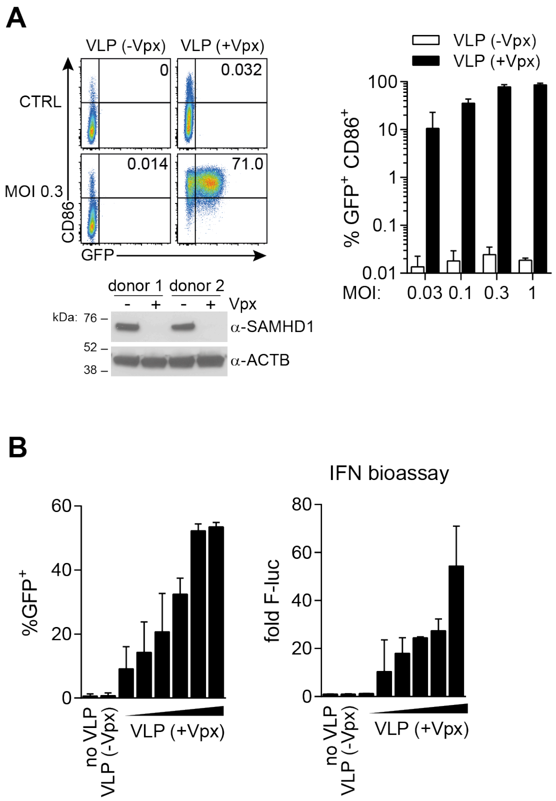

Figure S1, related to Figure 2. Vpx-mediated degradation of SAMHD1 leads to productive infection and activation of human DCs.

(A and B) Human MDDCs were co-infected for 48 h with FGLenti and Vpx-containing (+Vpx) or control (-Vpx) VLPs.

(A) Flow cytometric analysis of GFP (infectivity) and CD86 (activation) expression. Representative FACS plots are shown. The numbers represent percentages of GFP^+ $CD86^+$ cells. A quantification of GFP^+ $CD86^+$ MDDCs for increasing MOI's of FGLenti-RT^{WT} is shown in the right graph. Western blots to validate Vpx-mediated degradation of SAMHD1 are shown for MDDCs from two donors. β-ACTIN (ACTB) was used as a loading control.

(B) MDDCs were infected with FGLenti-RT^{WT} (MOI 0.3) and increasing doses of Vpx-containing VLPs. (left) Flow cytometric analysis of GFP expression (infectivity). (right) IFN in cell culture supernatants was measured by bioassay.

Data are representative of 3 independent experiments and represent mean ± SD (n = 4).

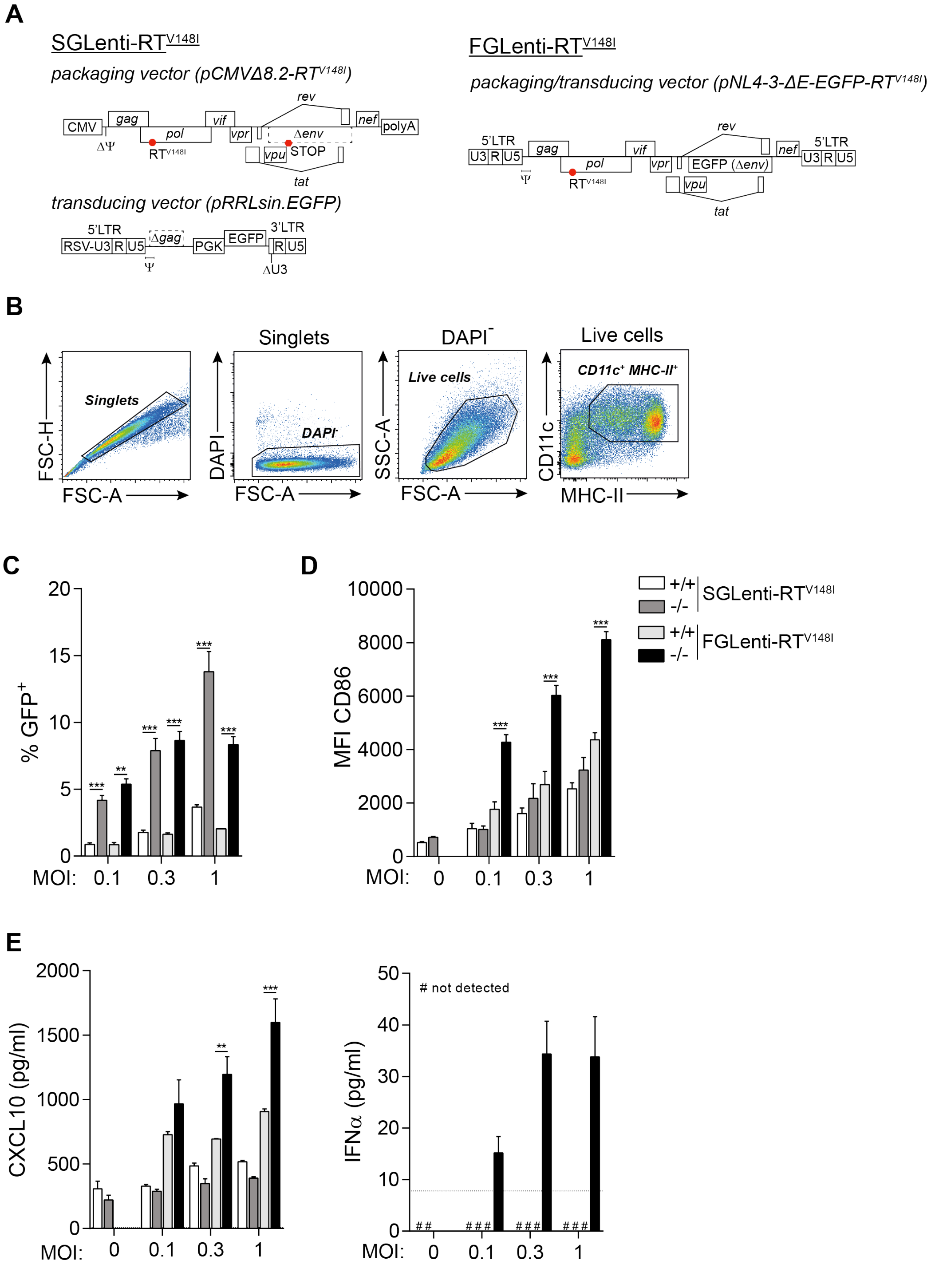

Figure S2, related to Figure 2. Infection with first-generation (FGLenti-RT^{V148I}) but not second-generation (SGLenti-RT^{V148I}) lentivirus induces BMMC activation.

(A) Schematic of the HIV-1-based viruses used in this study. The transducing vector of the second-generation lentivirus (pRRLsin.EGFP) lacks all HIV-1 proteins, contains an HIV-1/RSV hybrid 5'LTR and self-inactivating LTRs. The packaging vector (pCMVΔ8.2 RT^{V148I}) delivers all viral proteins *in trans* in the producer HEK293T cell line. In contrast, the first-generation lentivirus contains a full-length HIV-1 genome, in which the *Env* gene is replaced by *EGFP*. During virus production in HEK293T cells, the viral genome and all viral proteins derive from

one vector (pNL4-3-ΔE-EGFP-RT^{V148I}). Psi depicts the viral packaging signal. CMV and PGK are promoter sequences derived from cytomegalovirus and murine *Pgk1*. Red circles indicate the position of the V148I mutation, which decreases the binding of RT to dNTPs. All lentiviruses in this study were pseudotyped with VSV-G.
(B) The gating strategy applied in Figure 2A is shown. Only single and live cells ($DAPI^{-}$) were included to define the $CD11c^{+}$ $MHC\text{-}II^{+}$ population in BMMC cultures.
(C-E) Wild type (+/+) or *Samhd1*$^{-/-}$ (-/-) BMMCs were infected for 48 h with different MOIs of first-generation (FGlenti-RT^{V148I}) or second-generation lentivirus (SGlenti-RT^{V148I}) (n = 4).
(C) Infectivity was measured by flow cytometry. The percentage of GFP^{+} cells is shown after gating on $CD11c^{+}$ $MHC\text{-}II^{+}$ cells.
(D) Cells surface expression of CD86 was assessed by flow cytometry. The CD86 median fluorescence intensity (MFI) is shown after gating on $CD11c^{+}$ $MHC\text{-}II^{+}$ cells.
(E) CXCL10 and IFNα in cell culture supernatants were measured by ELISA.
Data are representative of 3 independent experiments. Data in C-E represent mean ± SD. **$p<0.01$, ***$p<0.001$; 2-WAY ANOVA.

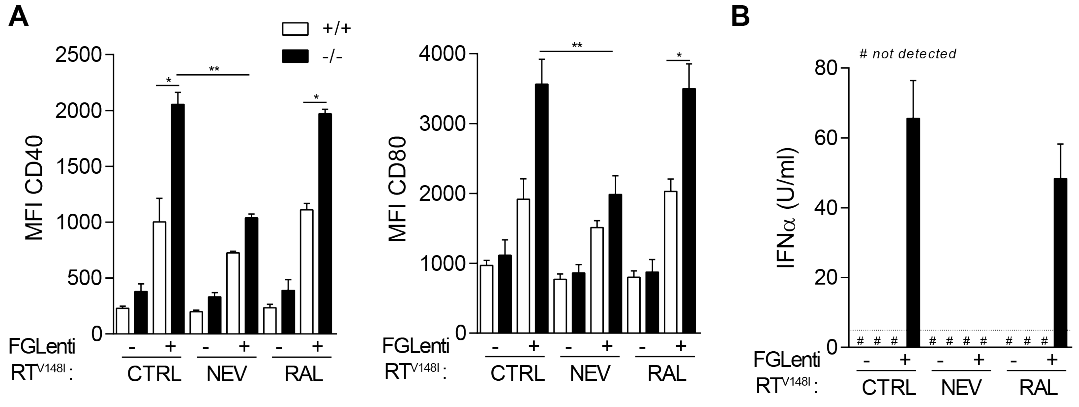

Figure S3, related to Figure 3. Sensing of first-generation lentivirus in *Samhd1*$^{-/-}$ BMMCs requires reverse transcription but not integration.

(A-B) Wild type (+/+) or SAMHD1-deficient (-/-) BMMCs were infected for 48 h in the presence of nevirapine (NEV) or raltegravir (RAL) (n = 4).

(A) CD40 and CD80 cell surface expression was assessed on CD11c^{+} MHC-II^{+} cells as in Figure 2B.

(B) IFNα in cell culture supernatants was measured by ELISA (n = 4).

Data are representative of 3 independent experiments. Data represent mean ± SD. *p < 0.05, **p<0.01; 2-WAY ANOVA.

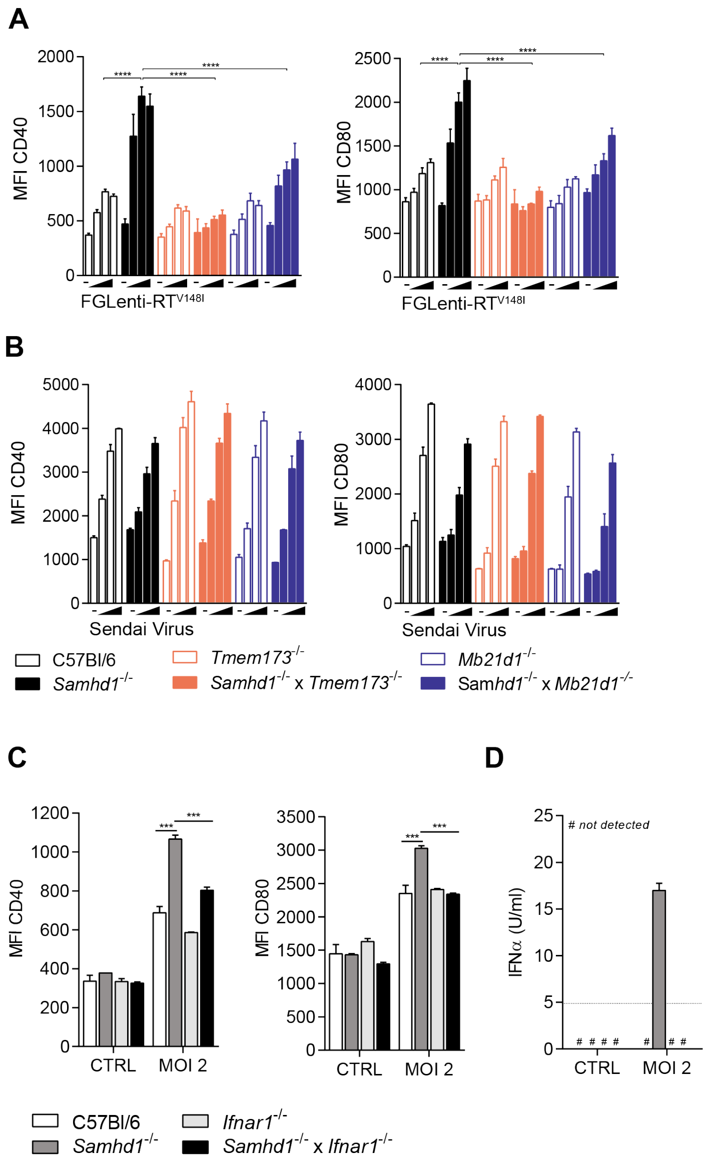

Figure S4, related to Figure 4. BMMC activation by first-generation lentivirus is dependent on cGAS, STING and IFN signalling.

(A-D) BMMCs of the indicated genotypes were infected for 48 h with FGLenti-RT^{V148I} or SeV. *Tmem173*, *Mb21d1* and *Ifnar1* are the genes encoding STING, cGAS and IFNAR, respectively. (A) CD40 and CD80 cell surface expression was assessed as in Figure 2B. Wedges represent increasing MOIs (0.1, 0.3 and 1) of FGLenti-RT^{V148I} (n = 4).

(B) CD40 and CD80 cell surface expression was assessed as in Figure 2B. Wedges represent increasing MOIs (0.02, 0.1 and 0.5) of Sendai virus (n = 2).

(C) CD40 and CD80 cell surface expression was assessed as in Figure 2B (n = 2).

(D) IFNα in cell culture supernatants was measured by ELISA (n = 2).

Data are representative of 3 independent experiments. Data represent mean ± SD. ***p<0.001, ****p<0.0001; 2-WAY ANOVA.

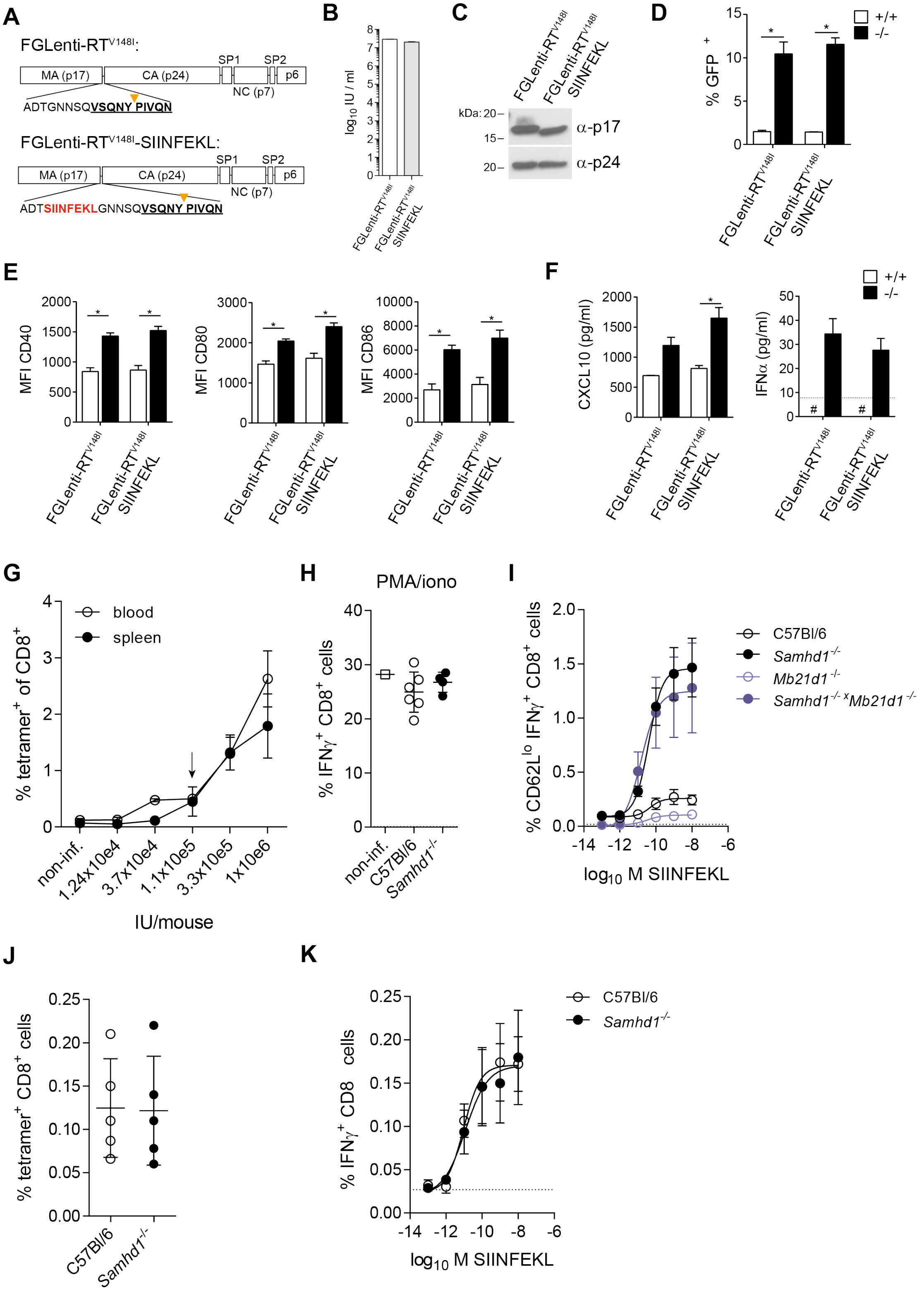

Figure S5, related to Figure 5. Characterization of SIINFEKL-expressing first-generation lentivirus (FGLenti-RT^{V148I} SIINFEKL).

(A) Schematic of the gag polypeptide of FGLenti-RT^{V148I} SIINFEKL. The $OVA_{257-264}$ (SIINFEKL) peptide was inserted at the C-terminus of matrix (MA). The protease cleavage sequence between MA and capsid (CA) is highlighted in bold and the proteolytic site is indicated by a yellow triangle.
(B) Viral titres in supernatants collected 72 h after transfection from HEK293T producer cells were determined by infection of fresh HEK293T and analysis of GFP^{+} cells by flow cytometry (n = 3).
(C) Western blot for CA (p24) and MA (p17) using protein lysates from purified virus preparations.
(D-F) Wild type (+/+) or *Samhd1*$^{-/-}$ (-/-) BMMCs were infected for 48 h with FGlenti-RT^{V148I} or FGLenti-RT^{V148I} SIINFEKL (MOI=0.3) (n = 4).
(D) Infectivity was measured as in Figure 2A.
(E) CD40, CD80 and CD86 cell surface expression was assessed as in Figure 2B.
(F) CXCL10 and IFNα in cell culture supernatants were measured by ELISA.
(G) Wild type (C57Bl/6, n = 2) mice were intravenously infected with increasing infectious units (IU) of FGLenti-RT^{V148I} SIINFEKL. Percentages of H2-Kb-SIINFEKL tetramer^{+} CD8 T cells present in the blood and spleen after 10 days of infection are shown.
(H) Quantification of IFNγ-expressing splenic CD8 T cells 6 h after PMA/ionomycin stimulation (C57Bl/6, n = 6 and *Samhd*1$^{-/-}$, n = 5).
(I) Mice of the indicated genotypes (*Mb21d1* encodes cGAS) were infected for 10 days with FGLenti-RT^{V148I} SIINFEKL. Spleen cells were stimulated for 6 hours with SIINFEKL peptide (10^{-13} to 10^{-8} M) prior to intracellular IFNγ staining and analysis by flow cytometry. Percentages of IFNγ^{+} activated (CD62^{lo}) CD8 T cells are shown.
(J-K) Wild type (C57Bl/6, n = 5) and *Samhd1*$^{-/-}$ mice (n = 5) were injected intramuscularly with 10μg OVA and 10μg cGAMP. (J) Quantification of H2-Kb-SIINFEKL tetramer^{+} CD8 T cells present in the spleen 10 days after challenge. Each dot represents an individual mouse. (K) Intracellular IFNγ staining of spleen cells from (J) after 6 hours of SIINFEKL peptide (10^{-13} to 10^{-8} M) re-stimulation. Percentages of IFNγ^{+} CD8 T cells are shown. The dashed line represents the fraction of IFNγ^{+} CD8 T cells responding to peptide in a mouse injected with PBS.
Data are representative of 2 (B-H) or 1 (I-K) independent experiment(s). Data in (B), (D-K) represent mean ± SD. *$p < 0.05$; unpaired t-test (B, D-H, J) and non-linear 4PL sigmoidal curve fitting (I, K).

Supplemental Tables

Table S1: oligonucleotides used for lentivirus plasmid cloning

**Plasmid**	**Lentivirus**	**Oligonucleotide**	**Sequence: 5' to 3'**
pNL4-3-deltaE-EGFP-RT^{V148I}	FGLenti-RT^{V148I}	Fwd-left-ApaI	ATTGCAGGGCCCCTAGGAAAAAGGGCT
		Rvs-left-V148I	CCATCCCTGTGGAAGGATATTGTACTGATATCT
		Fwd-right-V148I	AGATATCAGTACAATATCCTTCCACAGGGATGG
		Rvs-right-EcoRI	GTTGCAGAATTCTTATTATGGCTTCCACTCC
pNL4-3-deltaE-EGFP-RT^{V148I} SIINFEKL	FGLenti-RT^{V148I} SIINFEKL	Fwd-left-AatII	CCACCTGACGTCTAAGAAACCATTA
		Rvs-left-SIINFEKL	CAGCTTCTCGAAGTTGATGATGCTTGTGTCAGCTG CTGCTTGCTGTGCCTT
		Fwd-right-SIINFEKL	AGCATCATCAACTTCGAGAAGCTGGGAAACAACA GCCAGGTCAGCCAAAATTAC
		Rvs-right-ApaI	TCCTAGGGGCCCTGCAATTTTTGGCTA

Table S2: Plasmid combinations for lentivirus production

**Lentivirus**	**Plasmids**
FGLenti	pNL4-3-deltaE-EGFP and pMD2.G
FGLenti-RT^{V148I}	pNL4-3-deltaE-EGFP-RT^{V148I} and pMD2.G
FGLenti-RT^{V148I} SIINFEKL	pNL4-3-deltaE-EGFP-RT^{V148I} SIINFEKL and pMD2.G
SGLenti-RT^{V148I}	pRRLsin.EGFP, pCMVΔ8.2 RTV148I and pMD2.G
VLP(-Vpx)	pSIV4+ and pMD2.G
VLP(+Vpx)	pSIV3+ and pMD2.G

Table S3: TaqMan probes (Applied Biosystems)

**Gene**	**Assay Probe ID**
Ifi44	Mm00505670_m1
Ifit1	Mm00515153_m1
Ifit2	Mm00492606_m1
Oasl1	Mm00455081_m1
Isg15	Mm01705338_s1
Gapdh	Cat. no. 4352932E
EGFP	Mr04329676_mr
Tfrc	Cat. no. 4458366

Table S4: antibodies used for Western blot

**Antigen**	**Supplier**	**Host**	**Cat. number**	**Dilution**
HIV-1 p17	NIH AIDS Reagent Program	Rabbit polyclonal	4811	1:2,000
HIV-1 p24	Advanced Bioscience Laboratories	Mouse monoclonal	4313	1:5,000
Human SAMHD1	Abcam	Mouse monoclonal	Ab67820	1:1,000
Mouse SAMHD1	In house (Rehwinkel et al., 2013)	Rabbit polyclonal		1:100,000
Beta-actin	Sigma Aldrich	Mouse monoclonal (HRP-coupled)	A3854	1:100,000
ISG15	A. Pichlmair	Rabbit polyclonal		1:2,000

Table S5: antibodies used for flow cytometry

Cell surface marker	Supplier	Clone
Anti-human CD86	eBioscience	IT2.2
CD11c	eBioscience	N418
MHC-II	eBioscience	M5/114.15.2
CD80	eBioscience	16-10A1
CD86	eBioscience	GL1
SIINFEKL-H-2Kb	eBioscience	25-D1.16
CD62L	eBioscience	MEL-14
B220	eBioscience	RA3-6B2
CD40	Biolegend	3/23
IFNγ	BD Biosciences	XMG1.2

Supplemental Experimental Procedures

Plasmids and reagents

Nevirapine (#4666), raltegravir (#11680) and the pNL4-3-deltaE-EGFP plasmid (#11100; FGLenti) were obtained through the NIH AIDS Reagent Program, Division of AIDS, NIAID, NIH. pNL4-3-deltaE-EGFP was from Drs. Haili Zhang, Yan Zhou, and Robert Siliciano (Zhang et al., 2004). Plasmids for FGLenti-RT^{V148I} and FGLenti-RT^{V148I} SIINFEKL were cloned using pNL4-3-deltaE-EGFP as a template *via* overlap PCR using the oligo's listed in table S1. pMD2.G, pRRLsin.EGFP (SGLenti) and pCMVΔ8.2-RT^{V148I} have been described before (Rehwinkel et al., 2013). pSIV3+ and pSIV4+ are described in (Negre et al., 2000). IFNα A/D was purchased from Sigma Aldrich. SeV (Cat. No. VR-907) was purchased from ATCC.

Cells

BMMCs, BMDMs and human MDDCs were cultured in RPMI (Sigma Aldrich) supplemented with 10% heat inactivated FCS, 2 mM L-Gln, 100 U/ml penicillin, 0.1 mg/ml streptomycin and 50 μM 2-mercaptoethanol. For BMMCs and BMDMs bone marrow was cultured with 20 ng/ml GM-CSF (Peprotech) or 20% L929 supernatant. Medium was partially refreshed at day 2 and replaced entirely on day 3. Cultures contained on average 60-70% $CD11c^+$ $MHC\text{-}II^+$ cells or > 95% $CD11b^+$ $F4/80^+$ macrophages. For detection of the spontaneous IFN response, BMDMs were harvested at day 5 and cultured for an additional 7 days in RPMI medium supplemented with 20% L929 supernatant. Human MDDCs were differentiated for 5 days from $CD14^+$ monocytes with 40 ng/ml GM-CSF and 40 ng/ml IL-4. Monocytes were purified from total PBMCs using MACS separation columns and CD14 microbeads (Miltenyi). The cultures contained > 95% $DC\text{-}SIGN^+$ cells. TLA HEK293T cells (Open Biosystems) were grown in DMEM (Sigma Aldrich) supplemented with 10% heat inactivated FCS and 2 mM L-Gln. All cells were grown at 37°C and 5% CO_2.

Detection of IFN and CXCL10

Mouse IFNα was detected by ELISA. Anti-mouse IFNα antibody (clone F18, Hycult Biotech) and anti-mouse IFNα antibody (PBL Assay Science, 32100) were used as capturing and detection antibodies and IFNα (Hycult Biotech, HC1040b) as a standard. Alternatively, IFNα was measured by LumiKine IFNα ELISA (Invivogen). CXCL10 ELISA was from eBioscience. The HEK293-ISRE-luciferase based bioassay described in (Bridgeman et al., 2015) was used for the detection of human IFN. Human IFNα2 (R&D Systems) was used as a standard.

qPCR and RT-qPCR

Cells were lysed in RLT buffer, homogenized using a Qiaschredder (Qiagen) and purified using RNeasy columns with DNase I digestion (Qiagen). Tissues were homogenized with glass beads (Sigma Aldrich) in TRIzol (Thermo Fisher) on a FastPrep F120 instrument (Thermo Fisher). After chloroform phase-separation, RNA (aqueous phase) was further purified using RNeasy columns. cDNA synthesis was performed with SuperScript II reverse transcriptase (Thermo Fisher) and random hexamer primers (Ambion). For DNA extraction, cells were lysed in AL-buffer and incubated with proteinase K at 56°C for 10 min and purified using DNeasy blood and tissue columns (Qiagen). 15 ng of input material was amplified using TaqMan universal PCR or Taqman Genotyping Master Mix (for *Tfrc*) on a 7800 real-time PCR system (Applied Biosystems). Expression data were normalized to *Gapdh* (mRNA) or *Tfrc* (cDNA) and analyzed by the comparative C_t method. The TaqMan probes (Applied Biosystems) used in this study are listed in table S3.

Western blot and FACS

Cells and virus preparations were lysed in 25mM Tris.HCl pH 7.4, 150mM NaCl, 2mM EDTA, 1% Igepal CA-630 (Sigma Aldrich) and 5% glycerol and protease inhibitor cocktail (Cell Signalling). Protein lysates were cleared by centrifugation at 16,000g for 10 min. Antibodies used for Western blot are listed in table S4. Anti-rabbit or anti-mouse antibodies coupled to HRP were from GE Healthcare Life Science. Anti-mouse SAMHD1 was described in (Rehwinkel et al., 2013). Antiserum to HIV-1 p17 (4811) was obtained through the NIH AIDS Reagent Program, Division of AIDS, NIAID, NIH from Dr. Paul Spearman (Varthakavi et al., 1999). For FACS analysis, BMMCs were detached with Accutase (Sigma Aldrich). Blood was subjected to red blood lysis (ACK-buffer, Sigma Aldrich). Spleens were digested with 100 μg/ml collagenase type IV (Worthington) and 20 μg/ml DNase I (Roche). Cells were stained in FACS buffer (PBS, 2mM EDTA, 1%FCS). 1 μg/ml DAPI (Sigma Aldrich) or Live/Dead Fixable Violet (Molecular Probes) was used to exclude dead cells. Data were acquired on Beckmann Coulter CyAn

or BD Biosciences LSRFortessa flow cytometers. The antibodies used for FACS are listed in supplementary table 5. H-2K^b-SIINFEKL tetramer was a gift from V. Cerundolo.

Supplemental References

Negre, D., Mangeot, P.E., Duisit, G., Blanchard, S., Vidalain, P.O., Leissner, P., Winter, A.J., Rabourdin-Combe, C., Mehtali, M., Moullier, P., *et al.* (2000). Characterization of novel safe lentiviral vectors derived from simian immunodeficiency virus (SIVmac251) that efficiently transduce mature human dendritic cells. Gene therapy *7*, 1613-1623.

Varthakavi, V., Browning, P.J., and Spearman, P. (1999). Human immunodeficiency virus replication in a primary effusion lymphoma cell line stimulates lytic-phase replication of Kaposi's sarcoma-associated herpesvirus. Journal of virology *73*, 10329-10338.

Zhang, H., Zhou, Y., Alcock, C., Kiefer, T., Monie, D., Siliciano, J., Li, Q., Pham, P., Cofrancesco, J., Persaud, D., and Siliciano, R.F. (2004). Novel single-cell-level phenotypic assay for residual drug susceptibility and reduced replication capacity of drug-resistant human immunodeficiency virus type 1. Journal of virology *78*, 1718-1729.
