# Supplementary material for: Restriction by SAMHD1 Limits cGAS/STING-Dependent Innate and Adaptive Immune Responses to HIV-1
Source: Cell Rep. 2016 Jul 28;16(6):1492–501. doi: 10.1016/j.celrep.2016.07.002 (PMC4978700; doi:10.1016/j.celrep.2016.07.002)
Supplement: Document S2. Article plus Supplemental Information [file mmc2.pdf]

Cell Reports

Report

Restriction by SAMHD1 Limits cGAS/STING-Dependent Innate and Adaptive Immune Responses to HIV-1

Graphical Abstract

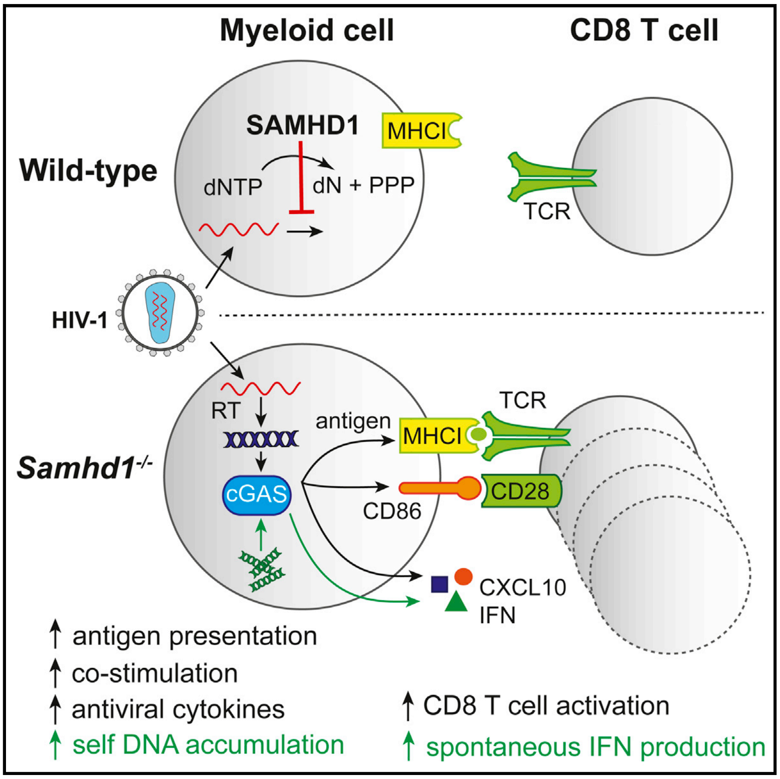

Highlights

- Spontaneous IFN production in SAMHD1-deficient cells requires cGAS and STING
- During HIV-1 infection, SAMHD1 limits activation of myeloid cells
- cGAS and STING detect HIV-1 infection in SAMHD1-deficient cells and induce IFN
- SAMHD1 prevents virus-specific CD8 T cell responses in vivo

Authors

Jonathan Maelfait, Anne Bridgeman, Adel Benlahrech, Chiara Cursi, Jan Rehwinkel

Correspondence

jan.rehwinkel@imm.ox.ac.uk

In Brief

Restriction factors defend infected cells against viruses. Maelfait et al. find that SAMHD1, which blocks HIV-1 infection in myeloid cells, prevents innate sensing of infection via cGAS/STING. Furthermore, SAMHD1 curtails virus-specific T cell responses in vivo. Restriction by SAMHD1, therefore, limits subsequent innate and adaptive immune responses.

Maelfait et al., 2016, Cell Reports *16*, 1492–1501
August 9, 2016 © 2016 The Author(s).
http://dx.doi.org/10.1016/j.celrep.2016.07.002

CellPress

Cell Reports
Report

Restriction by SAMHD1 Limits cGAS/STING-Dependent Innate and Adaptive Immune Responses to HIV-1

Jonathan Maelfait,[1] Anne Bridgeman,[1] Adel Benlahrech,[2] Chiara Cursi,[1] and Jan Rehwinkel[1,*]
[1]Medical Research Council Human Immunology Unit, Weatherall Institute of Molecular Medicine and Radcliffe Department of Medicine, University of Oxford, Oxford OX3 9DS, UK
[2]Medical Research Council Human Immunology Unit, Weatherall Institute of Molecular Medicine and Nuffield Department of Medicine, University of Oxford, Oxford OX3 9DS, UK
*Correspondence: jan.rehwinkel@imm.ox.ac.uk
http://dx.doi.org/10.1016/j.celrep.2016.07.002

SUMMARY

SAMHD1 is a restriction factor for HIV-1 infection. *SAMHD1* mutations cause the autoinflammatory Aicardi-Goutières syndrome that is characterized by chronic type I interferon (IFN) secretion. We show that the spontaneous IFN response in SAMHD1-deficient cells and mice requires the cGAS/STING cytosolic DNA-sensing pathway. We provide genetic evidence that cell-autonomous control of lentivirus infection in myeloid cells by SAMHD1 limits virus-induced production of IFNs and the induction of costimulatory markers. This program of myeloid cell activation required reverse transcription, cGAS and STING, and signaling through the IFN receptor. Furthermore, SAMHD1 reduced the induction of virus-specific cytotoxic T cells in vivo. Therefore, virus restriction by SAMHD1 limits the magnitude of IFN and T cell responses. This demonstrates a competition between cell-autonomous virus control and subsequent innate and adaptive immune responses, a concept with important implications for the treatment of infection.

INTRODUCTION

Virus infection in mammalian hosts is controlled by a variety of mechanisms operating at different levels. These include cell-intrinsic restriction systems, innate immune sensors that signal for the induction of an antiviral state, and cellular and adaptive immune responses. How these different branches of the antiviral response work together is important for successful immunity. The role of pattern-recognition receptors that sense infection for the development of subsequent immune responses has been well documented (Medzhitov, 2009). However, less is known about how virus control by restriction factors is linked with innate and adaptive immune responses.

Restriction factors have been studied in particular detail for HIV-1 and include APOBEC3G, TRIM5α, tetherin, and Mx2 (Rehwinkel, 2014; Simon et al., 2015). Another HIV-1 restriction factor is SAMHD1, a deoxynucleoside triphosphate (dNTP) triphosphohydrolase that depletes the intracellular pool of dNTPs and thereby prevents HIV-1 reverse transcription in some cell types (Ayinde et al., 2012). Additional mechanisms by which SAMHD1 might restrict infection have been proposed and include degradation and/or binding of viral nucleic acids (Ballana and Esté, 2015).

Several studies suggested that SAMHD1-deficient cells produce elevated levels of type I interferons (IFNs) in response to HIV-1 infection. Indirect evidence for this idea stems from experiments using Vpx, a viral accessory protein encoded by HIV-2, but not HIV-1. Vpx targets SAMHD1 for proteasomal degradation (Hrecka et al., 2011; Laguette et al., 2011). Depletion of SAMHD1 by Vpx in cultured human cells not only facilitates HIV-1 infection but also results in the induction of an antiviral response (Manel et al., 2010). In addition, cells from patients with *SAMHD1* mutations or cells in which SAMHD1 is depleted by RNAi produce more IFNs during HIV-1 infection (Berger et al., 2011; Puigdomènech et al., 2013). Subsequent work identified a role for cytosolic DNA sensing by cGAS and STING in IFN induction in Vpx-treated cells (Gao et al., 2013; Lahaye et al., 2013). Furthermore, SAMHD1 depletion in vitro in human dendritic cells (DCs) by Vpx delivery or RNAi enhances DC activation and antigen presentation upon HIV-1 infection and facilitates T cell responses in co-culture models (Ayinde et al., 2015). However, the interpretation of these data is complicated by the possibility that Vpx targets additional proteins apart from SAMHD1 (Fujita et al., 2012; Reinhard et al., 2014), by genetic heterogeneity of patients' cells, and by recent results that failed to reproduce enhanced DC activation in HIV-1-infected cells depleted of SAMHD1 (Hertoghs et al., 2015). In vivo data and genetic studies in knockout models interrogating the possible role of SAMHD1 in innate and adaptive immune responses to HIV-1 are currently lacking.

Mutations in human *SAMHD1* cause Aicardi-Goutières syndrome (AGS), a rare monogenic disorder resembling congenital virus infection and typified by early-onset brain disease (Rice et al., 2009). AGS patients spontaneously produce IFNs in the absence of infection with exogenous viruses (Crow and Manel, 2015). These observations suggest that SAMHD1 prevents the accumulation of endogenous nucleic acids that induce IFNs. Others and we previously reported spontaneous IFN production in *Samhd1*$^{-/-}$ mice and cells, mimicking the situation in AGS patients (Behrendt et al., 2013; Rehwinkel et al., 2013). The identity

This is an open access article under the CC BY license (http://creativecommons.org/licenses/by/4.0/).

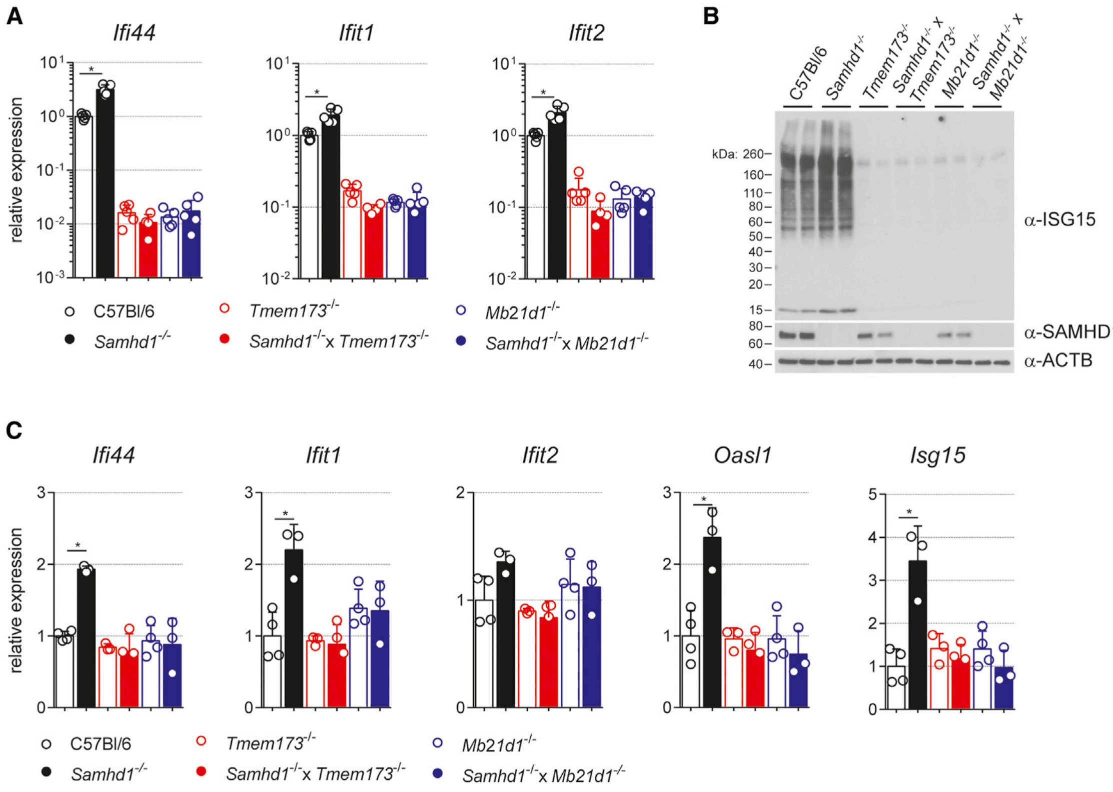

Figure 1. Loss of SAMHD1 Triggers a Spontaneous cGAS/STING-Dependent IFN Response

(A and B) BMDMs of the indicated genotypes were cultured for 12 days.

(A) mRNA expression of the indicated ISGs by RT-qPCR. Data are presented as fold changes compared to the average of wild-type (C57Bl/6) samples. Each open circle represents mean gene expression from two BMDM cultures from one mouse (n = 5).

(B) Western blot for ISG15, SAMHD1, and β-ACTIN (ACTB) using protein lysates from BMDMs. High-molecular-weight signals represent ISGylated proteins.

(C) mRNA expression of the indicated ISGs by RT-qPCR. Data are presented as fold changes compared to the mean of wild-type samples. Open circles represent gene expression values from individual 6-month-old mice. At least three mice were analyzed per genotype.

Data in (A) and (B) are representative of two independent experiments. Data in (A) and (C) represent mean ± SD (*$p < 0.05$, Student's t test).

and source of the cellular IFN-stimulatory nucleic acid and the signaling pathway activated by it are important open questions (Roers et al., 2016).

We show that cGAS and STING mediate chronic IFN production in *Samhd1*$^{-/-}$ mice and cells. We further show that SAMHD1-deficient myeloid cells produce heightened levels of IFNs in response to infection with VSV-G-pseudotyped HIV-1-derived lentiviruses and are activated more strongly. These effects required reverse transcription and were nullified in cells from *Samhd1*$^{-/-}$ mice also lacking cGAS, STING, or IFNAR. We also demonstrate that the induction of antigen-specific T cell responses is limited by SAMHD1 in vivo. Therefore, virus control by SAMHD1 at the level of the infected cell limits innate and adaptive immunity.

RESULTS

The Spontaneous IFN Response in *Samhd1*$^{-/-}$ Cells and Mice Requires cGAS and STING

AGS is a prototypical interferonopathy, a group of diseases characterized by chronic and pathologic IFN production (Crow and Manel, 2015). Mutation of *SAMHD1* or one of at least six other genes, including *TREX1* and *RNASEH2A*-C, causes AGS (Crow and Manel, 2015). Others and we previously showed that *Samhd1*$^{-/-}$ mice and cells display a spontaneous IFN signature, although SAMHD1-deficient animals do not develop autoimmunity (Behrendt et al., 2013; Rehwinkel et al., 2013). Given that SAMHD1's enzymatic function is to degrade dNTPs, the building blocks of DNA, we hypothesized that cytosolic DNA sensing might account for IFN induction in the absence of SAMHD1.

To test this, we first cultured bone marrow-derived macrophages (BMDMs) from SAMHD1-cGAS (*Mb21d1*$^{-/-}$) or SAMHD1-STING (*Tmem173*$^{-/-}$) double-knockout mice. Consistent with previous observations, *Samhd1*$^{-/-}$ BMDMs expressed higher mRNA levels of the interferon-stimulated genes (ISGs) *Ifi44*, *Ifit1*, and *Ifit2* compared to wild-type cells (Figure 1A). However, the expression of these ISGs was not increased in cells lacking both SAMHD1 and STING or cGAS compared to single-knockout control cells (Figure 1A). It is noteworthy that basal ISG expression was reduced in cGAS- and STING-deficient cells. Wild-type BMDMs thus maintain basal expression of ISGs and this requires an intact cytosolic DNA-sensing pathway.

ISG15 is an IFN-inducible ubiquitin-like protein modifier with antiviral functions (Morales and Lenschow, 2013). *Samhd1*$^{-/-}$ cells expressed more ISG15 protein and contained increased

OPEN
ACCESS
CellPress

amounts of high-molecular-weight proteins, which were covalently modified by ISG15 (Figure 1B). Enhanced ISGylation was not observed in SAMHD1-deficient BMDMs that did not express cGAS or STING (Figure 1B).

To confirm these findings in vivo, we analyzed spleens from 6-month-old *Samhd1*$^{-/-}$ mice for the expression of several ISGs. The mRNA levels of *Ifi44*, *Ifit1*, *Oasl1*, and *Isg15* were significantly increased in *Samhd1*$^{-/-}$ spleens (Figure 1C). However, the expression of these genes was reduced to wild-type levels when SAMHD1 was knocked out together with cGAS or STING (Figure 1C). In sum, these data show that SAMHD1 prevents spontaneous engagement of the cGAS/STING pathway.

SAMHD1 Limits IFN Induction and Myeloid Cell Activation upon Lentivirus Infection

Vpx-mediated degradation of SAMHD1 enables productive infection of human DCs and relieves a block to IFN induction and DC activation (Figure S1) (Manel et al., 2010). To test this in a genetically tractable system, we generated bone marrow-derived myeloid cells (BMMCs) from wild-type and *Samhd1*$^{-/-}$ mice (Helft et al., 2015). Others and we previously showed that SAMHD1 restricts infection of these cells with HIV-1-based lentiviruses (Behrendt et al., 2013; Rehwinkel et al., 2013). Profound SAMHD1-dependent restriction both in vitro and in vivo was only observed when the *pol* gene carried a point mutation (V148I), which decreases the affinity of the viral RT for dNTPs (Rehwinkel et al., 2013). Second-generation lentiviruses used in our previous study (SGLenti-RT^{V148I}), which have a minimal genome encoding only EGFP (Figure S2A), were efficiently restricted by SAMHD1, but they did not induce BMMC activation or IFN secretion (Figures S2C–S2E) (Rehwinkel et al., 2013).

We therefore infected murine BMMCs with first-generation lentiviruses (FGlenti) that induce IFN in human monocyte-derived dendritic cells (MDDCs) (Figure S1). To allow for potent restriction by SAMHD1, we introduced the V148I point mutation into the *pol* gene of FGLenti (Figure S2A). Infection of BMMCs with both first- and second-generation lentiviruses was equally suppressed by SAMHD1 (Figures 2A and S2C). However, only FGLenti-RT^{V148I} induced cell surface expression of the co-stimulatory molecules CD40, CD80, and CD86 and secretion of IFNα and the chemokine CXCL10, and these effects were significantly elevated in SAMHD1-deficient cells (Figures 2B, 2C, S2D, and S2E). *Ifit1* and *Ifi44* mRNA and ISG15 protein expression and conjugation were enhanced in SAMHD1-deficient BMMCs (Figures 2D and 2E). Taken together, these data provide direct genetic evidence that SAMHD1-mediated restriction impedes detection of lentivirus infection in myeloid cells.

Viral cDNA Synthesis Is Required for Myeloid Cell Activation

To determine at which step in the viral life cycle HIV-1 is sensed in SAMHD1-deficient myeloid cells, we treated BMMCs with the RT inhibitor nevirapine or the integrase inhibitor raltegravir during infection with FGLenti-RT^{V148I}. Both compounds inhibited infection (Figure 3A), but only inhibition of reverse transcription prevented BMMC activation and CXCL10 and IFNα production (Figures 3B, 3C, S3A, and S3B). SAMHD1 may restrict HIV by multiple mechanisms, including by preventing cDNA synthesis and by degrading incoming viral genomic RNA. We found that HIV-1 cDNA accumulated over 24 hr at 10-fold higher levels in *Samhd1*$^{-/-}$ BMMCs compared to wild-type cells (Figure 3D). In contrast, viral RNA levels measured at 4 hr post-infection were not affected by SAMHD1 (Figure 3E). At this time point we observed higher induction of *Ifnb* and *Ifit1* mRNAs in SAMHD1-deficient BMMCs (Figure 3F). These results indicate that viral RNAs do not trigger the IFN response at this time point, which instead depends on reverse transcription and correlates in magnitude with reverse transcription products.

Lentivirus-Induced Myeloid Cell Activation and IFN Induction Are Dependent on cGAS/STING and IFN Signaling

Given the requirement for reverse transcription, we tested whether the cytosolic DNA-sensing pathway detects lentivirus infection in SAMHD1-deficient myeloid cells. SAMHD1-cGAS and SAMHD1-STING double-knockout BMMCs were equally or slightly better infected by FGLenti-RT^{V148I} than *Samhd1*$^{-/-}$ cells (Figure 4A). BMMC activation, the secretion of CXCL10 and IFNα, and ISG15 protein expression and conjugation were all greatly reduced in *Samhd1*$^{-/-}$ BMMCs lacking cGAS or STING (Figures 4B–4D and S4A). Neither BMMC activation nor IFNα production were affected by loss of SAMHD1, cGAS, or STING upon infection with Sendai virus (SeV), which is sensed via the RIG-I pathway (Kato et al., 2006) (Figures 4E, 4F, and S4B). Next, we assessed the role of IFN signaling in activation of SAMHD1-deficient myeloid cells by infecting SAMHD1-type I IFN receptor (*Ifnar1*$^{-/-}$) double-knockout BMMCs with FGlenti-RT^{V148I}. Despite the fact that loss of the type I IFN receptor further increased susceptibility to FGlenti-RT^{V148I} compared to SAMHD1 single-knockout cells (Figure 4G), BMMC activation and antiviral responses in these cells were impaired (Figures 4H–4J, S4C, and S4D). In sum, these observations provide genetic evidence that cGAS, STING, and signaling through the type I IFN receptor are required for SAMHD1-dependent myeloid cell activation upon lentivirus infection.

SAMHD1 Reduces the Antiviral CD8 T Cell Response In Vivo

Given that SAMHD1 curtails the induction of IFN and the activation of myeloid cells in vitro, we tested whether SAMHD1 limits adaptive immune responses. We fused the OVA$_{257–264}$ peptide SIINFEKL, a model antigen for CD8 T cells (Carbone and Bevan, 1989), to the C terminus of the matrix protein (Figure S5A). The resulting FGLenti-RT^{V148I} SIINFEKL virus behaved identically to the parental virus in terms of virus production, gag processing, infectivity, and BMMC activation (Figures S5B–S5F). Furthermore, SIINFEKL presentation by H-2Kb was enhanced in *Samhd1*$^{-/-}$ cells (Figure 5A).

Next we compared the antigen-specific CD8 T cell response in wild-type and *Samhd1*$^{-/-}$ animals. Compared to wild-type mice, infection of *Samhd1*$^{-/-}$ animals resulted in 4- and 10-fold increased tetramer-positive CD8 T cell populations in blood and spleens, respectively (Figures 5B, 5C, and S5G). Spleens of *Samhd1*$^{-/-}$ mice contained more CD8 T cells producing IFNγ upon SIINFEKL re-stimulation than those of wild-type controls (Figures 5D and 5E), but not after phorbol 12-myristate

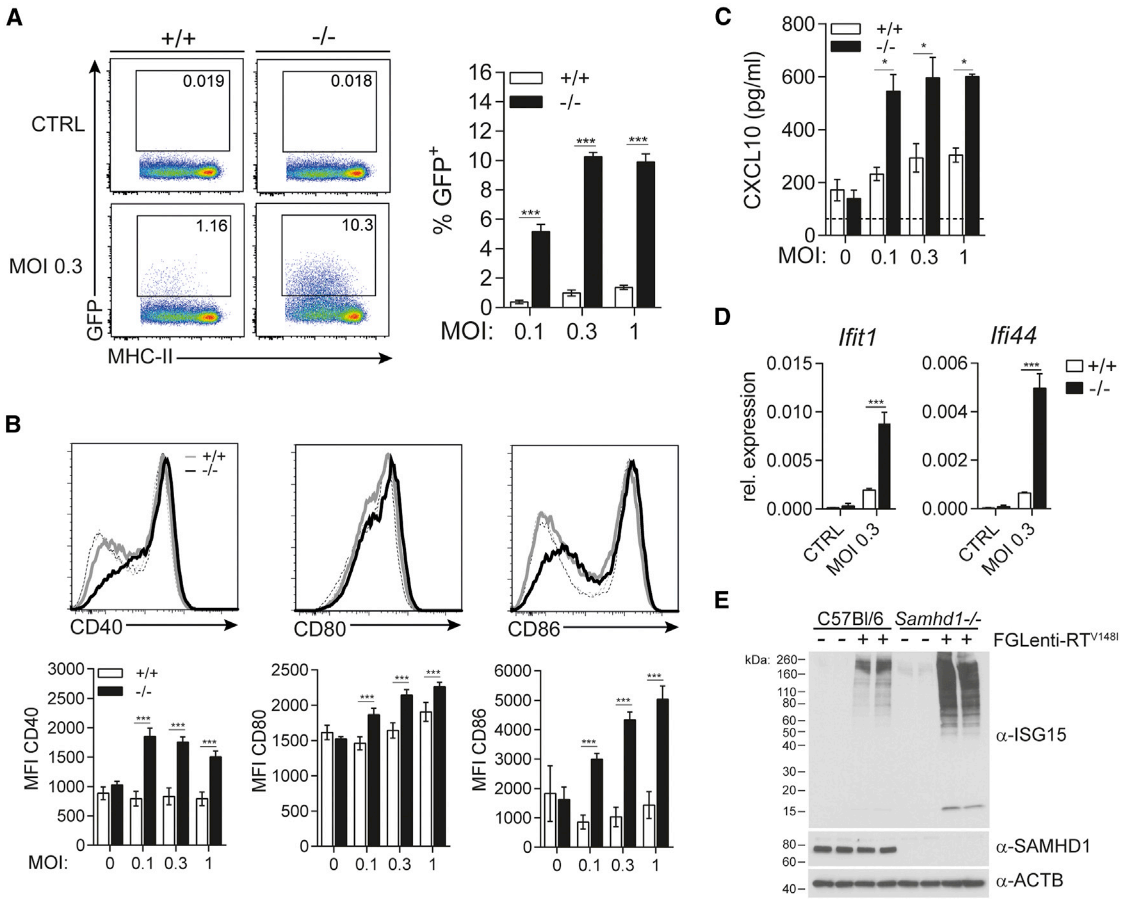

Figure 2. SAMHD1 Limits Infection of BMMCs with First-Generation Lentiviruses and Prevents Innate Sensing

(A–E) Wild-type (+/+) or *Samhd1*$^{-/-}$ (–/–) BMMCs were infected for 48 hr with FGlenti-RT^{V148I} using the indicated MOIs.

(A) Infection was assessed by flow cytometry. BMMC cultures were gated on CD11c^{+} MHC-II^{+} cells (see Figure S2B). The fraction of GFP^{+} cells was then determined. Representative FACS plots (left) and percentage of GFP^{+} cells are shown (right) (n = 4).

(B) Activation of CD11c^{+} MHC-II^{+} BMMCs was analyzed by cell surface staining for CD40, CD80, and CD86. Upper panels depict representative histograms of non-infected (dashed lines) and infected (solid lines, MOI = 0.3) cells. CD40, CD80, and CD86 median fluorescence intensity (MFI) of cells infected with different MOIs is quantified in the bottom graphs (n = 4).

(C) CXCL10 in cell culture supernatant was measured by ELISA (n = 4).

(D) *Ifit1* and *Ifi44* mRNA expression was analyzed by RT-qPCR in bulk BMMC cultures (n = 4).

(E) ISG15, SAMHD1, and β-ACTIN (ACTB) were analyzed by western blot using protein lysates from bulk BMMC cultures (MOI = 0.3).

Data are representative of three (A–C) and two (D and E) independent experiments. Data in (A)–(D) represent mean ± SD (*p < 0.05 and ***p < 0.001, two-way ANOVA). See also Figures S1 and S2.

13-acetate (PMA) and ionomycin stimulation (Figure S5H). SAMHD1-cGAS double-knockout mice mounted a similar antigen-specific CD8 T cell response compared to mice lacking only SAMHD1 (Figure S5I). This indicates that the increased antigen load in *Samhd1*$^{-/-}$ animals determines the magnitude of the CD8 T cell response in this model. To exclude direct effects of SAMHD1 on the CD8 T cell repertoire or T cell activation, we injected *Samhd1*$^{-/-}$ and wild-type mice with OVA protein using cGAMP as an adjuvant (Li et al., 2013). This resulted in comparable SIINFEKL-specific CD8 T cell responses in wild-type and *Samhd1*$^{-/-}$ mice (Figures S5J and S5K). We conclude that SAMHD1 limits lentivirus-specific T cell responses to lentiviral infection in vivo.

DISCUSSION

Our study provides genetic and in vivo data that the restriction factor SAMHD1 does not operate in conjunction with other innate and adaptive immune responses to ensure control of lentivirus infection. Instead, SAMHD1 limits the induction of IFNs, ISGs and co-stimulatory molecules as well as the amounts of antigen presented by infected myeloid cells in vitro. Furthermore, SAMHD1 limits the magnitude of the CD8 T cell response induced in vivo. Cell-autonomous virus restriction by SAMHD1, therefore, competes with sensors of infection and T cell responses. This antagonistic relationship between SAMHD1 and innate and adaptive immune responses is of importance for

OPEN
ACCESS
CellPress

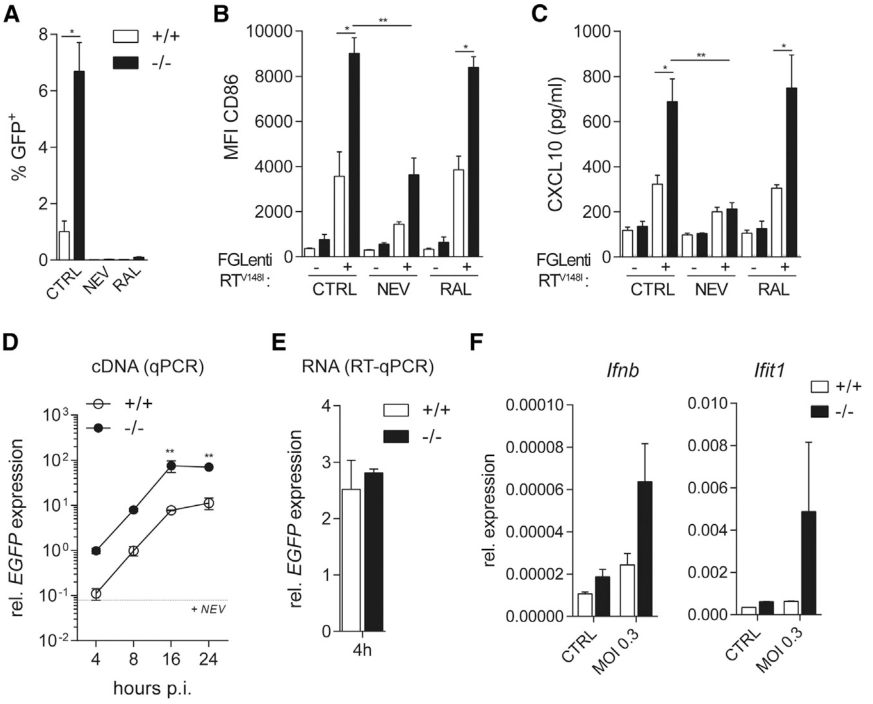

Figure 3. Sensing of First-Generation Lentivirus in *Samhd1*$^{-/-}$ BMMCs Requires the Accumulation of Reverse Transcription Products

(A–F) Wild-type (+/+) or *Samhd1*$^{-/-}$ (−/−) BMMCs were infected with FGlenti-RT^{V148I} (MOI = 0.3) in the presence of nevirapine (NEV) or raltegravir (RAL) (A–C).

(A) Infectivity was measured as in Figure 2A 48 hr after infection (n = 4).

(B) Cell surface expression of CD86 was assessed as in Figure 2B 48 hr after infection (n = 4).

(C) CXCL10 in cell culture supernatants was measured by ELISA 48 hr after infection (n = 4).

(D) cDNA production was measured by qPCR. Total DNA was extracted from BMMCs at different hours post-infection (p.i.). The dashed line depicts *EGFP* expression in wild-type BMMCs at 16 hr p.i. in the presence of NEV as a control for plasmid contamination of the virus preparation (n = 2).

(E) Viral genomic RNA was measured by RT-qPCR using total RNA extracted from BMMCs at 4 hr p.i.

(F) *Ifnb* and *Ifit1* mRNA expression was analyzed by RT-qPCR in bulk BMMC cultures at 4 hr p.i. (n = 2).

Data are representative of three (A–C) or two (D–F) independent experiments. Data in (A)–(F) represent mean ± SD (*$p < 0.05$ and **$p < 0.01$; one-way ANOVA, A–C; two-way ANOVA, D and F; unpaired t test, E). See also Figure S3.

our understanding of lentiviral pathogenicity, and it may provide therapeutic opportunities to control viral infections.

It is interesting to ask whether this concept also applies to other restriction factors. Indeed, a recent study showed that cultured DCs inactivate TRIM5α's function by redirecting the protein to the cell nucleus, and it suggested that this reflects an evolutionary trade-off in DCs, in which restriction is minimized to allow for more efficient sensing (Portilho et al., 2016). However, other restriction factors do not compete with but rather facilitate subsequent innate and adaptive immune responses. APOBEC3 is required for efficient production of antibodies in retrovirus-infected mice (Santiago et al., 2008, 2010; Smith et al., 2011), and human APOBEC3G facilitates T cell responses (Casartelli et al., 2010). Tetherin promotes NK and CD8 T cell responses in a mouse model of retrovirus infection (Li et al., 2014). Interestingly, some restriction factors, including TRIM5α and tetherin, also operate as sensors of infection and induce NF-κB (Galão et al., 2012; Pertel et al., 2011). Viral restriction factors thus differentially impact the host's immune responses, and it is likely that this complexity is due to host-pathogen co-evolution.

A first hint at the possibility that SAMHD1 prevents sensing of virus infection came from its implication in AGS, a rare autoinflammatory disease characterized by chronic IFN production (Rice et al., 2009). Here we show that the spontaneous IFN response in *Samhd1*$^{-/-}$ cells and mice requires cGAS and STING. Together with our previous observation that *Samhd1*$^{-/-}$ cells respond normally to stimulation with exogenous DNA or STING agonists (Rehwinkel et al., 2013), these data suggest that an unusual endogenous DNA accumulates in SAMHD1-deficient cells and engages cGAS. This mirrors the situation in TREX1- and RNASEH2-deficient cells (Roers et al., 2016).

We further report that IFN production in lentivirus-infected *Samhd1*$^{-/-}$ myeloid cells requires cGAS and STING. Induction of antiviral responses was dependent upon reverse transcription, implicating viral reverse-transcribed DNA as the cGAS trigger. These data provide genetic evidence that lentivirus-induced innate immune responses are dependent on cytosolic DNA sensing. As reported for human DCs (Manel et al., 2010), we find that first-generation lentiviruses encoding a full genome and second-generation viruses with a minimal genome differ in their abilities to induce IFN. It has been suggested that newly expressed gag unmasks non-integrated cDNA in the DC cytosol, making it available for detection by cGAS (Lahaye et al., 2013; Manel et al., 2010). Indeed, gag is expressed by first-generation lentiviruses, but not by second-generation viruses. Specific nucleic acid sequences or secondary structures only present in the full HIV-1 genome may also, perhaps in conjunction with additional host proteins, contribute to IFN induction (Herzner et al., 2015; Yoh et al., 2015).

To study the impact of lentivirus restriction by SAMHD1 on cytotoxic T cell responses in an in vivo model, we generated a lentivirus expressing the OVA peptide SIINFEKL. We found that infected *Samhd1*$^{-/-}$ myeloid cells better presented this model antigen. Enhanced antigen presentation also had been observed in HIV-1-infected human DCs upon SAMHD1 depletion with Vpx or RNAi, and these treatments resulted in stronger T cell responses in co-culture models (Ayinde et al., 2015; Manel et al., 2010). These in vitro studies, however, mostly relied on HIV-1-specific memory T cells, which have altered requirements for activation (Berard and Tough, 2002). It was, therefore, important

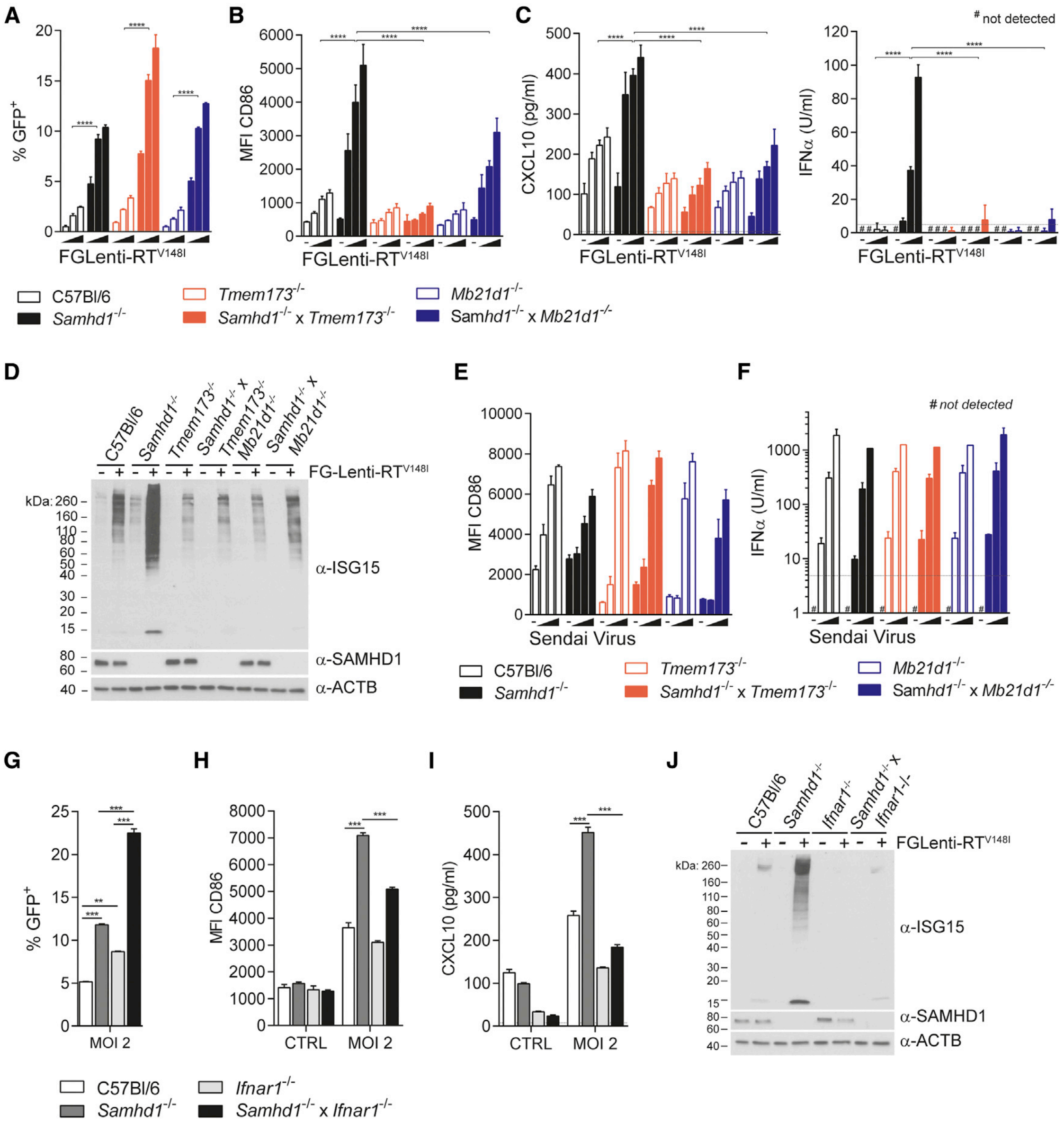

Figure 4. BMMC Activation by First-Generation Lentivirus Is Dependent on cGAS, STING, and IFN Signaling

(A–J) BMMCs of the indicated genotypes were infected for 48 hr with FGLenti-RT^{V148I} or SeV. Wedges in (A)–(C), (E), and (F) represent increasing MOIs of FGLenti-RT^{V148I} (0.1, 0.3, and 1) or SeV (0.02, 0.1, and 0.5). *Tmem173*, *Mb21d1*, and *Ifnar1* are the genes encoding STING, cGAS, and type I IFN receptor.

(A) Infectivity was measured as in Figure 2A (n = 4).

(B) Cell surface expression of CD86 was assessed as in Figure 2B (n = 4).

(C) CXCL10 and IFNα in cell culture supernatants were measured by ELISA (n = 4).

(D) ISG15, SAMHD1, and β-ACTIN (ACTB) were analyzed by western blot (MOI = 0.3).

(E) Cell surface expression of CD86 was assessed as in Figure 2B (n = 2).

(F) IFNα in cell culture supernatants was measured by ELISA (n = 2).

(G) Infectivity was measured as in Figure 2A (n = 2).

(H) Cell surface expression of CD86 was assessed as in Figure 2B (n = 2).

(I) CXCL10 in cell culture supernatants was measured by ELISA (n = 2).

(J) ISG15, SAMHD1, and β-ACTIN (ACTB) were analyzed by western blot.

Data are representative of three independent experiments. Data in (A)–(C) and (E)–(I) represent mean ± SD (**$p < 0.01$, ***$p < 0.001$, and ****$p < 0.0001$; two-way ANOVA, A–C, E, F, H, and I; one-way ANOVA, G). See also Figure S4.

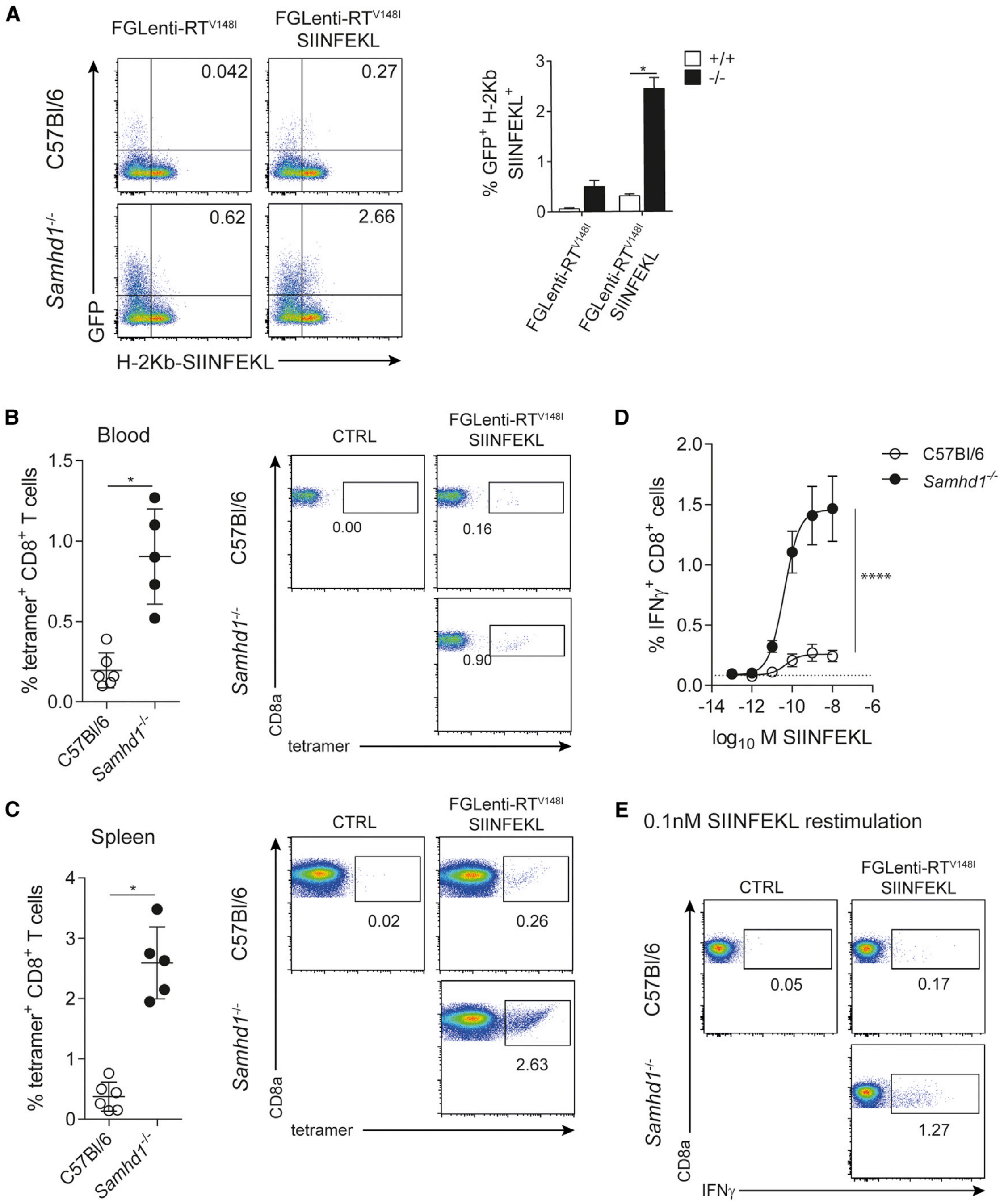

Figure 5. SAMHD1 Prevents Lentivirus-Specific CD8 T Cell Responses In Vivo

(A) GFP expression (infectivity) and antigen presentation (using a H2-Kb SIINFEKL-specific antibody) were analyzed by flow cytometry in CD11c$^+$ MHC-II$^+$ BMMCs 48 hr after infection with FGlenti-RT^{V148I} or FGLenti-RT^{V148I} SIINFEKL (MOI = 0.3). Representative FACS plots are shown on the left and a quantification of infected cells presenting SIINFEKL on MHC-I (GFP$^+$ H2-Kb SIINFEKL$^+$) is shown in the right panel (n = 4).

(B–E) Wild-type (C57Bl/6, n = 6) and *Samhd1*$^{-/-}$ mice (n = 5) were intravenously infected with FGLenti-RT^{V148I} SIINFEKL (100,000 IU/mouse).

(B and C) Quantification of H2-Kb-SIINFEKL tetramer$^+$ CD8 T cells in the blood 9 days p.i. (B) and in the spleen 10 days p.i. (C). Each dot represents an individual mouse. Representative FACS plots are shown on the right. Numbers indicate percentages of tetramer$^+$ CD8 T cells.

(D) Intracellular IFNγ staining of spleen cells from (C) after 6 hr of SIINFEKL peptide (10^{-13}–10^{-8} M) re-stimulation. Percentages of IFNγ$^+$ CD8 T cells are shown. The dashed line represents the fraction of IFNγ$^+$ CD8 T cells responding to peptide in an uninfected mouse.

(E) Representative FACS plots after re-stimulation with 10^{-10} M SIINFEKL. Numbers indicate percentages of IFNγ$^+$ CD8 T cells.

Data are representative of two independent experiments. Data in (A)–(D) represent mean ± SD (*p < 0.05 and ****p < 0.0001; unpaired t test, A–C; non-linear 4PL sigmoidal curve fitting, D). See also Figure S5.

to establish an in vivo model in which naive T cells are primed. Notably, we found that antigen-specific CD8 T cell responses were up to 10-fold stronger in *Samhd1*$^{-/-}$ mice compared to wild-type controls upon infection with the SIINFEKL-encoding lentivirus. It is interesting to ask whether this is due to enhanced antigen levels and/or to augmented co-stimulation and secretion of cytokines by antigen-presenting cells. We found that the SIINFEKL-specific T cell response was similarly enhanced in infected *Samhd1*$^{-/-}$ and *Samhd1*$^{-/-}$; *Mb21d1*$^{-/-}$ animals. Thus, in this setting, increased antigen levels are largely responsible for the effect of SAMHD1 on the T cell response. However, H-2Kb-bound SIINFEKL elicits strong T cell responses due to its exceptionally high affinity for the T cell receptor, and little signals 2 and 3 (co-stimulation and cytokines) are required for the SIINFEKL response (Denton et al., 2011). It will therefore be interesting to test other antigens in the future.

An interesting comparison can be made with HIV-2, which overcomes SAMHD1-mediated restriction by encoding the antagonist Vpx (Hrecka et al., 2011; Laguette et al., 2011). In line with our conclusion that SAMHD1 limits immune responses, in vitro infection of DCs with HIV-2 results in potent IFN induction (Lahaye et al., 2013). In humans, HIV-2 infection in comparison with HIV-1 infection is characterized by better immune control, and fewer HIV-2-infected individuals progress to disease (Nyamweya et al., 2013; Rowland-Jones and Whittle, 2007). It is possible that these effects are due in part to relief of SAMHD1-mediated restriction in HIV-2-infected cells, allowing for better innate and adaptive immune responses, and that HIV-1 has become the more successful pathogen by allowing itself to be restricted by SAMHD1. Consistent with this notion are observations in HIV-1 elite controllers. The expression level of SAMHD1 is reduced in DCs from elite controllers; these cells mount enhanced IFN responses to infection and are superior in vitro in stimulating CD8 T cells (Martin-Gayo et al., 2015).

These observations, together with the genetic evidence presented here for an inhibitory role of SAMHD1 on immune responses, suggest a strategy for the development of an efficient HIV-1 vaccine in the form of a replication-deficient virus that circumvents restriction by SAMHD1. These could be achieved either by inclusion of Vpx or by the development of small molecule inhibitors of SAMHD1. Pharmacological inhibition of SAMHD1 also could be useful in therapeutic approaches aimed at eradicating latent HIV-1. These strategies often rely on re-activation of latent provirus and subsequent killing of the virus by the immune system, which we predict would be more effective in the absence of SAMHD1 (Margolis and Hazuda, 2013). In sum, our work sheds light on the in vivo interplay among different branches of the antiviral immune system, and these insights have important implications for controlling infection.

EXPERIMENTAL PROCEDURES

Mice

All mice were on the C57Bl/6 background. *Samhd1*$^{-/-}$ mice were described previously (Rehwinkel et al., 2013). *Ifnar1*$^{-/-}$ mice were a gift from C. Reis e Sousa and were originally described in (Müller et al., 1994). *Tmem173*$^{-/-}$ (STING-deficient) mice were a gift from J. Cambier (Jin et al., 2011). *Mb21d1*$^{-/-}$ (cGAS knockout first) mice were described in Bridgeman et al. (2015). This work was performed in accordance with the UK Animals (Scientific Procedures) Act 1986 and institutional guidelines for animal care. This work was approved by a project license granted by the UK Home Office (PPL No. 40/3583) and also was approved by the Institutional Animal Ethics Committee Review Board at the University of Oxford.

Virus Production, Infection, and OVA-cGAMP Immunizations

All lentiviruses were produced in TLA HEK293T cells. Cells were transfected using Fugene6 (Promega) using the plasmid combinations listed in Table S2. All viruses were VSV-G pseudotyped. Medium was replaced the day after transfection, and 48 hr later supernatant was collected and filtered through a 0.45-μm polyethersulfone (PES) filter. Virus was concentrated by centrifugation over a 20% sucrose cushion at 50,000 × *g* for 2 hr. Viral titers were determined on TLA HEK293T cells by fluorescence-activated cell sorting (FACS) based on GFP expression. Target cells were infected in the presence of 8 μg/ml polybrene (Sigma-Aldrich). After 2 hr, the inoculum was washed away and replaced with fresh medium. For measuring viral cDNA and RNA, virus was incubated with 10 U/ml DNase I (Turbo DNase I, Thermo Fisher Scientific) for 30 min at 37°C in 1× reaction buffer diluted in RPMI. Then 10 μM nevirapine or raltegravir was added to the cells 30 min prior to infection. Mice were infected by intravenous injection of 100,000 IU of FGLenti-RT^{V148I} SIINFEKL (see Figure S5G for virus titration). For immunization with OVA and cGAMP, mice were anesthetized with isoflurane and injected intramuscularly with 10 μg 2′3′-cGAMP (Biolog, C 161) and 10 μg endotoxin-free OVA (Hyglos, 321000).

In Vitro Peptide Restimulation Assay

Spleen cells (2 × 10^6) were stimulated with SIINFEKL peptide (InvivoGen, 10^{-13}–10^{-8} M). After 1 hr, 10 μg/ml brefeldin A (Sigma-Aldrich) was added to the cells, and 5 hr later, cells were collected for intracellular IFNγ staining. Cells were stained for cell surface markers and intracellular molecules, and they were fixed and permeabilized in Cytofix/Cytoperm (BD Biosciences) according to the manufacturer's instructions.

Statistics

Statistical analysis was performed in GraphPad Prism v7.00 as detailed in the figure legends.

SUPPLEMENTAL INFORMATION

Supplemental Information includes Supplemental Experimental Procedures, five figures, and five tables and can be found with this article online at http://dx.doi.org/10.1016/j.celrep.2016.07.002.

AUTHOR CONTRIBUTIONS

J.M. and J.R. conceived the study, designed experiments, analyzed data, and wrote the manuscript. J.M. performed all experiments. A. Bridgeman, A. Benlahrech, and C.C. provided technical help and advice. All authors read and approved the final manuscript.

ACKNOWLEDGMENTS

The authors thank C. Reis e Sousa, A. Jackson, V. Cerundolo, U. Gileadi, P. Borrow, A. Armitage, and members of the J.R. lab for critical discussions and reading of the manuscript draft. This work was funded by the UK Medical Research Council (MRC core funding of the MRC Human Immunology Unit) and the Wellcome Trust (grant 100954). J.M. was a recipient of a European Molecular Biology Organization (EMBO) long-term postdoctoral fellowship and was supported by Marie Curie Actions (EMBOCOFUND2010, GA-2010-267146). *Tmem173*$^{-/-}$ mice were provided by J. Cambier and are subject to material transfer agreements. *Ifnar1*$^{-/-}$ mice were provided by C. Reis e Sousa. H-2K^{b}-SIINFEKL tetramer was a gift from V. Cerundolo. Anti-ISG15 was a gift from A. Pichlmair.

OPEN
ACCESS
CellPress

Received: March 18, 2016
Revised: May 20, 2016
Accepted: July 1, 2016
Published: July 28, 2016

REFERENCES

Ayinde, D., Casartelli, N., and Schwartz, O. (2012). Restricting HIV the SAMHD1 way: through nucleotide starvation. Nat. Rev. Microbiol. *10*, 675–680.

Ayinde, D., Bruel, T., Cardinaud, S., Porrot, F., Prado, J.G., Moris, A., and Schwartz, O. (2015). SAMHD1 limits HIV-1 antigen presentation by monocyte-derived dendritic cells. J. Virol. *89*, 6994–7006.

Ballana, E., and Esté, J.A. (2015). SAMHD1: at the crossroads of cell proliferation, immune responses, and virus restriction. Trends Microbiol. *23*, 680–692.

Behrendt, R., Schumann, T., Gerbaulet, A., Nguyen, L.A., Schubert, N., Alexopoulou, D., Berka, U., Lienenklaus, S., Peschke, K., Gibbert, K., et al. (2013). Mouse SAMHD1 has antiretroviral activity and suppresses a spontaneous cell-intrinsic antiviral response. Cell Rep. *4*, 689–696.

Berard, M., and Tough, D.F. (2002). Qualitative differences between naïve and memory T cells. Immunology *106*, 127–138.

Berger, A., Sommer, A.F., Zwarg, J., Hamdorf, M., Welzel, K., Esly, N., Panitz, S., Reuter, A., Ramos, I., Jatiani, A., et al. (2011). SAMHD1-deficient CD14+ cells from individuals with Aicardi-Goutières syndrome are highly susceptible to HIV-1 infection. PLoS Pathog. *7*, e1002425.

Bridgeman, A., Maelfait, J., Davenne, T., Partridge, T., Peng, Y., Mayer, A., Dong, T., Kaever, V., Borrow, P., and Rehwinkel, J. (2015). Viruses transfer the antiviral second messenger cGAMP between cells. Science *349*, 1228–1232.

Carbone, F.R., and Bevan, M.J. (1989). Induction of ovalbumin-specific cytotoxic T cells by in vivo peptide immunization. J. Exp. Med. *169*, 603–612.

Casartelli, N., Guivel-Benhassine, F., Bouziat, R., Brandler, S., Schwartz, O., and Moris, A. (2010). The antiviral factor APOBEC3G improves CTL recognition of cultured HIV-infected T cells. J. Exp. Med. *207*, 39–49.

Crow, Y.J., and Manel, N. (2015). Aicardi-Goutières syndrome and the type I interferonopathies. Nat. Rev. Immunol. *15*, 429–440.

Denton, A.E., Wesselingh, R., Gras, S., Guillonneau, C., Olson, M.R., Mintern, J.D., Zeng, W., Jackson, D.C., Rossjohn, J., Hodgkin, P.D., et al. (2011). Affinity thresholds for naive CD8+ CTL activation by peptides and engineered influenza A viruses. J. Immunol. *187*, 5733–5744.

Fujita, M., Nomaguchi, M., Adachi, A., and Otsuka, M. (2012). SAMHD1-dependent and -independent functions of HIV-2/SIV Vpx protein. Front Microbiol *3*, 297.

Galão, R.P., Le Tortorec, A., Pickering, S., Kueck, T., and Neil, S.J. (2012). Innate sensing of HIV-1 assembly by Tetherin induces NFκB-dependent proinflammatory responses. Cell Host Microbe *12*, 633–644.

Gao, D., Wu, J., Wu, Y.T., Du, F., Aroh, C., Yan, N., Sun, L., and Chen, Z.J. (2013). Cyclic GMP-AMP synthase is an innate immune sensor of HIV and other retroviruses. Science *341*, 903–906.

Helft, J., Böttcher, J., Chakravarty, P., Zelenay, S., Huotari, J., Schraml, B.U., Goubau, D., and Reis e Sousa, C. (2015). GM-CSF mouse bone marrow cultures comprise a heterogeneous population of CD11c(+)MHCII(+) macrophages and dendritic cells. Immunity *42*, 1197–1211.

Hertoghs, N., van der Aar, A.M., Setiawan, L.C., Kootstra, N.A., Gringhuis, S.I., and Geijtenbeek, T.B. (2015). SAMHD1 degradation enhances active suppression of dendritic cell maturation by HIV-1. J. Immunol. *194*, 4431–4437.

Herzner, A.M., Hagmann, C.A., Goldeck, M., Wolter, S., Kübler, K., Wittmann, S., Gramberg, T., Andreeva, L., Hopfner, K.P., Mertens, C., et al. (2015). Sequence-specific activation of the DNA sensor cGAS by Y-form DNA structures as found in primary HIV-1 cDNA. Nat. Immunol. *16*, 1025–1033.

Hrecka, K., Hao, C., Gierszewska, M., Swanson, S.K., Kesik-Brodacka, M., Srivastava, S., Florens, L., Washburn, M.P., and Skowronski, J. (2011). Vpx relieves inhibition of HIV-1 infection of macrophages mediated by the SAMHD1 protein. Nature *474*, 658–661.

Jin, L., Hill, K.K., Filak, H., Mogan, J., Knowles, H., Zhang, B., Perraud, A.L., Cambier, J.C., and Lenz, L.L. (2011). MPYS is required for IFN response factor 3 activation and type I IFN production in the response of cultured phagocytes to bacterial second messengers cyclic-di-AMP and cyclic-di-GMP. J. Immunol. *187*, 2595–2601.

Kato, H., Takeuchi, O., Sato, S., Yoneyama, M., Yamamoto, M., Matsui, K., Uematsu, S., Jung, A., Kawai, T., Ishii, K.J., et al. (2006). Differential roles of MDA5 and RIG-I helicases in the recognition of RNA viruses. Nature *441*, 101–105.

Laguette, N., Sobhian, B., Casartelli, N., Ringeard, M., Chable-Bessia, C., Ségéral, E., Yatim, A., Emiliani, S., Schwartz, O., and Benkirane, M. (2011). SAMHD1 is the dendritic- and myeloid-cell-specific HIV-1 restriction factor counteracted by Vpx. Nature *474*, 654–657.

Lahaye, X., Satoh, T., Gentili, M., Cerboni, S., Conrad, C., Hurbain, I., El Marjou, A., Lacabaratz, C., Lelièvre, J.D., and Manel, N. (2013). The capsids of HIV-1 and HIV-2 determine immune detection of the viral cDNA by the innate sensor cGAS in dendritic cells. Immunity *39*, 1132–1142.

Li, X.D., Wu, J., Gao, D., Wang, H., Sun, L., and Chen, Z.J. (2013). Pivotal roles of cGAS-cGAMP signaling in antiviral defense and immune adjuvant effects. Science *341*, 1390–1394.

Li, S.X., Barrett, B.S., Heilman, K.J., Messer, R.J., Liberatore, R.A., Bieniasz, P.D., Kassiotis, G., Hasenkrug, K.J., and Santiago, M.L. (2014). Tetherin promotes the innate and adaptive cell-mediated immune response against retrovirus infection in vivo. J. Immunol. *193*, 306–316.

Manel, N., Hogstad, B., Wang, Y., Levy, D.E., Unutmaz, D., and Littman, D.R. (2010). A cryptic sensor for HIV-1 activates antiviral innate immunity in dendritic cells. Nature *467*, 214–217.

Margolis, D.M., and Hazuda, D.J. (2013). Combined approaches for HIV cure. Curr. Opin. HIV AIDS *8*, 230–235.

Martin-Gayo, E., Buzon, M.J., Ouyang, Z., Hickman, T., Cronin, J., Pimenova, D., Walker, B.D., Lichterfeld, M., and Yu, X.G. (2015). Potent cell-intrinsic immune responses in dendritic cells facilitate HIV-1-specific T cell immunity in HIV-1 elite controllers. PLoS Pathog. *11*, e1004930.

Medzhitov, R. (2009). Approaching the asymptote: 20 years later. Immunity *30*, 766–775.

Morales, D.J., and Lenschow, D.J. (2013). The antiviral activities of ISG15. J. Mol. Biol. *425*, 4995–5008.

Müller, U., Steinhoff, U., Reis, L.F., Hemmi, S., Pavlovic, J., Zinkernagel, R.M., and Aguet, M. (1994). Functional role of type I and type II interferons in antiviral defense. Science *264*, 1918–1921.

Nyamweya, S., Hegedus, A., Jaye, A., Rowland-Jones, S., Flanagan, K.L., and Macallan, D.C. (2013). Comparing HIV-1 and HIV-2 infection: lessons for viral immunopathogenesis. Rev. Med. Virol. *23*, 221–240.

Pertel, T., Hausmann, S., Morger, D., Züger, S., Guerra, J., Lascano, J., Reinhard, C., Santoni, F.A., Uchil, P.D., Chatel, L., et al. (2011). TRIM5 is an innate immune sensor for the retrovirus capsid lattice. Nature *472*, 361–365.

Portilho, D.M., Fernandez, J., Ringeard, M., Machado, A.K., Boulay, A., Mayer, M., Müller-Trutwin, M., Beignon, A.S., Kirchhoff, F., Nisole, S., and Arhel, N.J. (2016). Endogenous TRIM5α function is regulated by SUMOylation and nuclear sequestration for efficient innate sensing in dendritic cells. Cell Rep. *14*, 355–369.

Puigdomènech, I., Casartelli, N., Porrot, F., and Schwartz, O. (2013). SAMHD1 restricts HIV-1 cell-to-cell transmission and limits immune detection in monocyte-derived dendritic cells. J. Virol. *87*, 2846–2856.

Rehwinkel, J. (2014). Mouse knockout models for HIV-1 restriction factors. Cell. Mol. Life Sci. *71*, 3749–3766.

Rehwinkel, J., Maelfait, J., Bridgeman, A., Rigby, R., Hayward, B., Liberatore, R.A., Bieniasz, P.D., Towers, G.J., Moita, L.F., Crow, Y.J., et al. (2013). SAMHD1-dependent retroviral control and escape in mice. EMBO J. *32*, 2454–2462.

Reinhard, C., Bottinelli, D., Kim, B., and Luban, J. (2014). Vpx rescue of HIV-1 from the antiviral state in mature dendritic cells is independent of the intracellular deoxynucleotide concentration. Retrovirology *11*, 12.

Rice, G.I., Bond, J., Asipu, A., Brunette, R.L., Manfield, I.W., Carr, I.M., Fuller, J.C., Jackson, R.M., Lamb, T., Briggs, T.A., et al. (2009). Mutations involved in Aicardi-Goutières syndrome implicate SAMHD1 as regulator of the innate immune response. Nat. Genet. *41*, 829–832.

Roers, A., Hiller, B., and Hornung, V. (2016). Recognition of endogenous nucleic acids by the innate immune system. Immunity *44*, 739–754.

Rowland-Jones, S.L., and Whittle, H.C. (2007). Out of Africa: what can we learn from HIV-2 about protective immunity to HIV-1? Nat. Immunol. *8*, 329–331.

Santiago, M.L., Montano, M., Benitez, R., Messer, R.J., Yonemoto, W., Chesebro, B., Hasenkrug, K.J., and Greene, W.C. (2008). Apobec3 encodes Rfv3, a gene influencing neutralizing antibody control of retrovirus infection. Science *321*, 1343–1346.

Santiago, M.L., Benitez, R.L., Montano, M., Hasenkrug, K.J., and Greene, W.C. (2010). Innate retroviral restriction by Apobec3 promotes antibody affinity maturation in vivo. J. Immunol. *185*, 1114–1123.

Simon, V., Bloch, N., and Landau, N.R. (2015). Intrinsic host restrictions to HIV-1 and mechanisms of viral escape. Nat. Immunol. *16*, 546–553.

Smith, D.S., Guo, K., Barrett, B.S., Heilman, K.J., Evans, L.H., Hasenkrug, K.J., Greene, W.C., and Santiago, M.L. (2011). Noninfectious retrovirus particles drive the APOBEC3/Rfv3 dependent neutralizing antibody response. PLoS Pathog. *7*, e1002284.

Yoh, S.M., Schneider, M., Seifried, J., Soonthornvacharin, S., Akleh, R.E., Olivieri, K.C., De Jesus, P.D., Ruan, C., de Castro, E., Ruiz, P.A., et al. (2015). PQBP1 is a proximal sensor of the cGAS-dependent innate response to HIV-1. Cell *161*, 1293–1305.

Cell Reports, Volume *16*

Supplemental Information

Restriction by SAMHD1 Limits cGAS/STING-Dependent Innate and Adaptive Immune Responses to HIV-1

Jonathan Maelfait, Anne Bridgeman, Adel Benlahrech, Chiara Cursi, and Jan Rehwinkel

Supplemental Figures

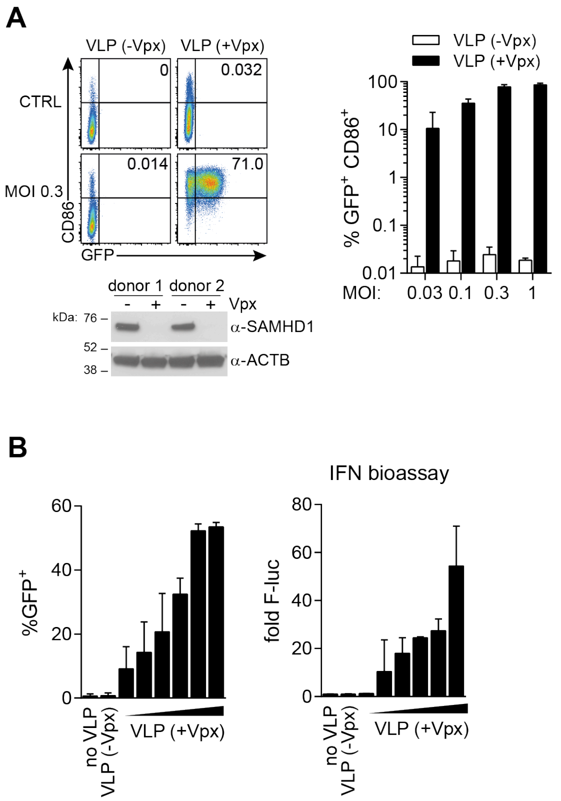

Figure S1, related to Figure 2. Vpx-mediated degradation of SAMHD1 leads to productive infection and activation of human DCs.

(A and B) Human MDDCs were co-infected for 48 h with FGLenti and Vpx-containing (+Vpx) or control (-Vpx) VLPs.

(A) Flow cytometric analysis of GFP (infectivity) and CD86 (activation) expression. Representative FACS plots are shown. The numbers represent percentages of GFP^+ $CD86^+$ cells. A quantification of GFP^+ $CD86^+$ MDDCs for increasing MOI's of FGLenti-RT^{WT} is shown in the right graph. Western blots to validate Vpx-mediated degradation of SAMHD1 are shown for MDDCs from two donors. β-ACTIN (ACTB) was used as a loading control.

(B) MDDCs were infected with FGLenti-RT^{WT} (MOI 0.3) and increasing doses of Vpx-containing VLPs. (left) Flow cytometric analysis of GFP expression (infectivity). (right) IFN in cell culture supernatants was measured by bioassay.

Data are representative of 3 independent experiments and represent mean ± SD (n = 4).

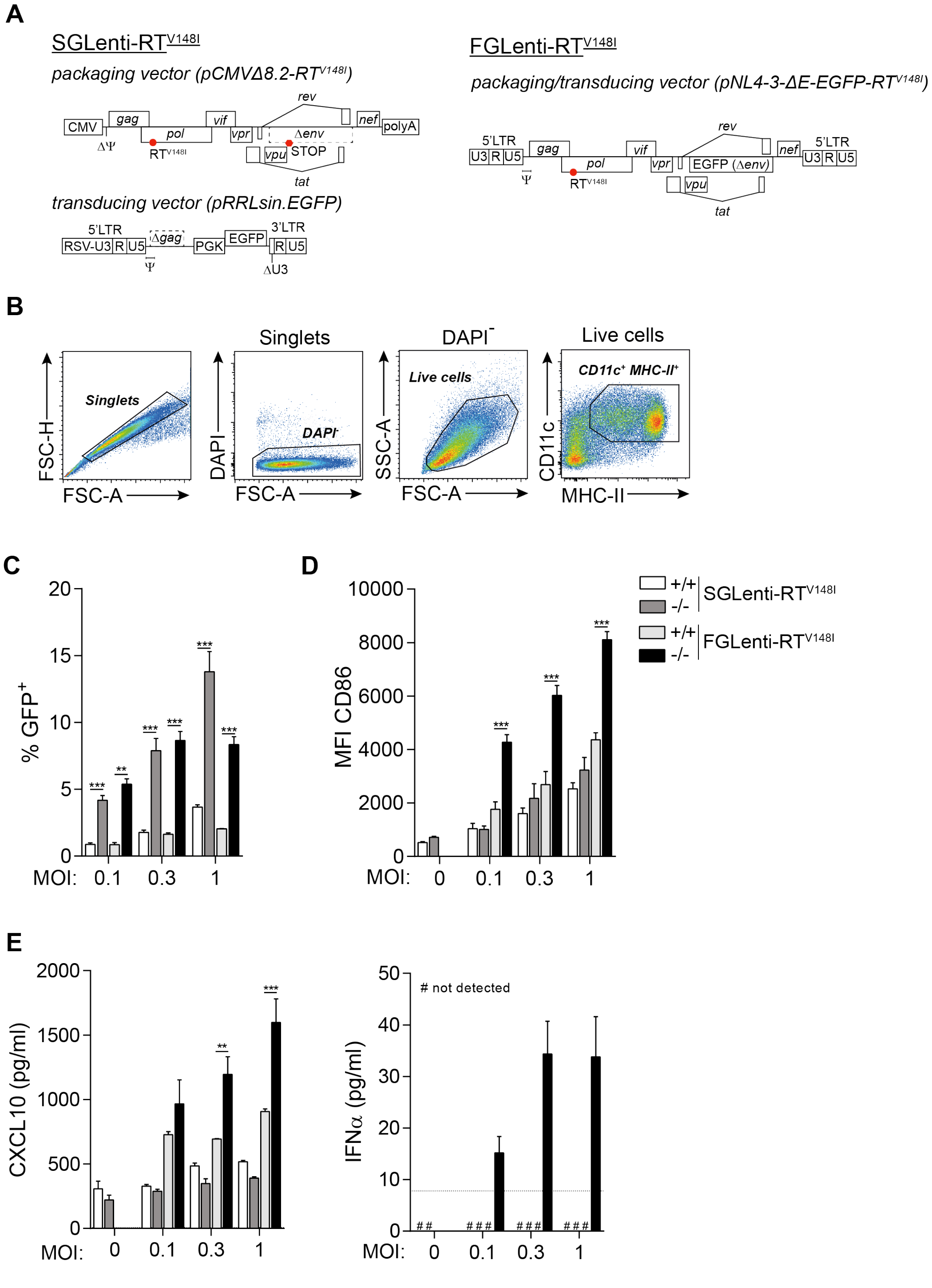

Figure S2, related to Figure 2. Infection with first-generation (FGLenti-RT^{V148I}) but not second-generation (SGLenti-RT^{V148I}) lentivirus induces BMMC activation.

(A) Schematic of the HIV-1-based viruses used in this study. The transducing vector of the second-generation lentivirus (pRRLsin.EGFP) lacks all HIV-1 proteins, contains an HIV-1/RSV hybrid 5'LTR and self-inactivating LTRs. The packaging vector (pCMVΔ8.2 RT^{V148I}) delivers all viral proteins *in trans* in the producer HEK293T cell line. In contrast, the first-generation lentivirus contains a full-length HIV-1 genome, in which the *Env* gene is replaced by *EGFP*. During virus production in HEK293T cells, the viral genome and all viral proteins derive from

one vector (pNL4-3-ΔE-EGFP-RT^{V148I}). Psi depicts the viral packaging signal. CMV and PGK are promoter sequences derived from cytomegalovirus and murine *Pgk1*. Red circles indicate the position of the V148I mutation, which decreases the binding of RT to dNTPs. All lentiviruses in this study were pseudotyped with VSV-G.
(B) The gating strategy applied in Figure 2A is shown. Only single and live cells (DAPI$^-$) were included to define the CD11c$^+$ MHC-II$^+$ population in BMMC cultures.
(C-E) Wild type (+/+) or *Samhd1*$^{-/-}$ (-/-) BMMCs were infected for 48 h with different MOIs of first-generation (FGlenti-RT^{V148I}) or second-generation lentivirus (SGlenti-RT^{V148I}) (n = 4).
(C) Infectivity was measured by flow cytometry. The percentage of GFP$^+$ cells is shown after gating on CD11c$^+$ MHC-II$^+$ cells.
(D) Cells surface expression of CD86 was assessed by flow cytometry. The CD86 median fluorescence intensity (MFI) is shown after gating on CD11c$^+$ MHC-II$^+$ cells.
(E) CXCL10 and IFNα in cell culture supernatants were measured by ELISA.
Data are representative of 3 independent experiments. Data in C-E represent mean ± SD. **p<0.01, ***p<0.001; 2-WAY ANOVA.

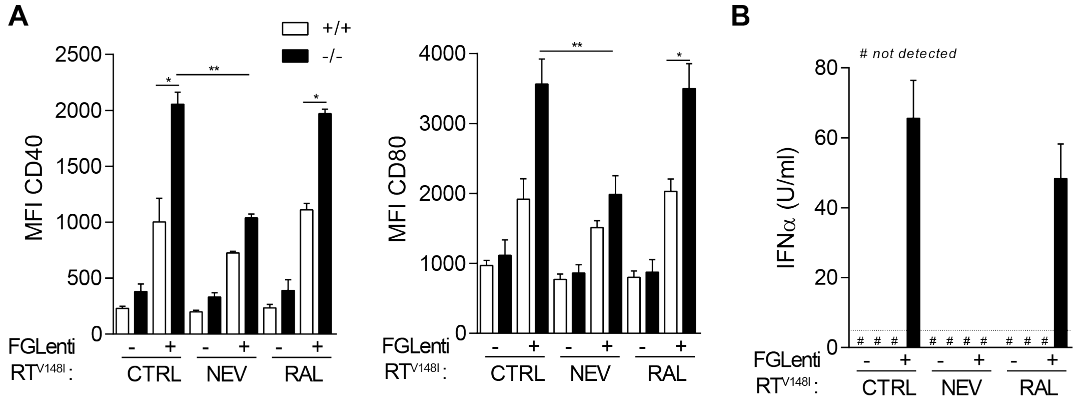

Figure S3, related to Figure 3. Sensing of first-generation lentivirus in *Samhd1*$^{-/-}$ BMMCs requires reverse transcription but not integration.

(A-B) Wild type (+/+) or SAMHD1-deficient (-/-) BMMCs were infected for 48 h in the presence of nevirapine (NEV) or raltegravir (RAL) (n = 4).

(A) CD40 and CD80 cell surface expression was assessed on CD11c^{+} MHC-II^{+} cells as in Figure 2B.

(B) IFNα in cell culture supernatants was measured by ELISA (n = 4).

Data are representative of 3 independent experiments. Data represent mean ± SD. *p < 0.05, **p<0.01; 2-WAY ANOVA.

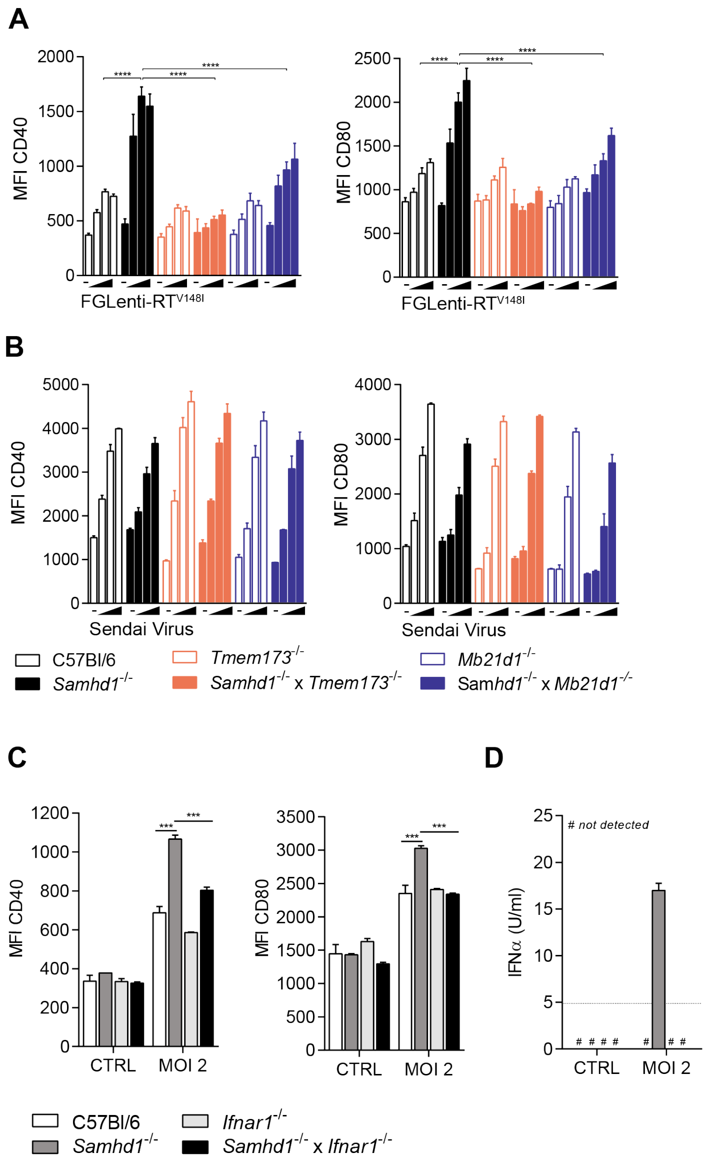

Figure S4, related to Figure 4. BMMC activation by first-generation lentivirus is dependent on cGAS, STING and IFN signalling.

(A-D) BMMCs of the indicated genotypes were infected for 48 h with FGLenti-RT^{V148I} or SeV. *Tmem173*, *Mb21d1* and *Ifnar1* are the genes encoding STING, cGAS and IFNAR, respectively. (A) CD40 and CD80 cell surface expression was assessed as in Figure 2B. Wedges represent increasing MOIs (0.1, 0.3 and 1) of FGLenti-RT^{V148I} (n = 4).

(B) CD40 and CD80 cell surface expression was assessed as in Figure 2B. Wedges represent increasing MOIs (0.02, 0.1 and 0.5) of Sendai virus (n = 2).

(C) CD40 and CD80 cell surface expression was assessed as in Figure 2B (n = 2).

(D) IFNα in cell culture supernatants was measured by ELISA (n = 2).

Data are representative of 3 independent experiments. Data represent mean ± SD. ***p<0.001, ****p<0.0001; 2-WAY ANOVA.

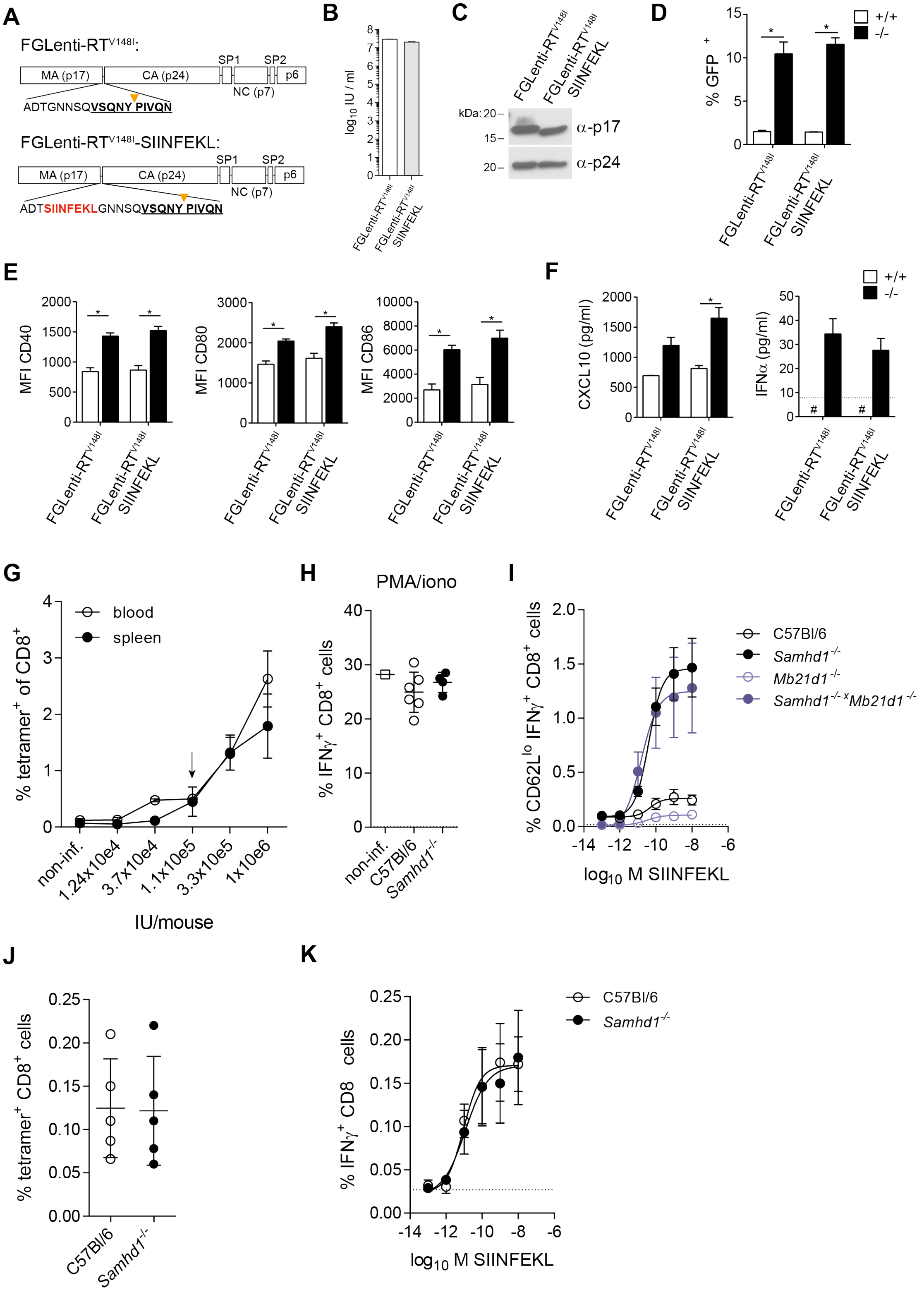

Figure S5, related to Figure 5. Characterization of SIINFEKL-expressing first-generation lentivirus (FGLenti-RT^{V148I} SIINFEKL).

(A) Schematic of the gag polypeptide of FGLenti-RT^{V148I} SIINFEKL. The $OVA_{257-264}$ (SIINFEKL) peptide was inserted at the C-terminus of matrix (MA). The protease cleavage sequence between MA and capsid (CA) is highlighted in bold and the proteolytic site is indicated by a yellow triangle.
(B) Viral titres in supernatants collected 72 h after transfection from HEK293T producer cells were determined by infection of fresh HEK293T and analysis of GFP^{+} cells by flow cytometry (n = 3).
(C) Western blot for CA (p24) and MA (p17) using protein lysates from purified virus preparations.
(D-F) Wild type (+/+) or *Samhd1*$^{-/-}$ (-/-) BMMCs were infected for 48 h with FGlenti-RT^{V148I} or FGLenti-RT^{V148I} SIINFEKL (MOI=0.3) (n = 4).
(D) Infectivity was measured as in Figure 2A.
(E) CD40, CD80 and CD86 cell surface expression was assessed as in Figure 2B.
(F) CXCL10 and IFNα in cell culture supernatants were measured by ELISA.
(G) Wild type (C57Bl/6, n = 2) mice were intravenously infected with increasing infectious units (IU) of FGLenti-RT^{V148I} SIINFEKL. Percentages of H2-Kb-SIINFEKL tetramer^{+} CD8 T cells present in the blood and spleen after 10 days of infection are shown.
(H) Quantification of IFNγ-expressing splenic CD8 T cells 6 h after PMA/ionomycin stimulation (C57Bl/6, n = 6 and *Samhd*1$^{-/-}$, n = 5).
(I) Mice of the indicated genotypes (*Mb21d1* encodes cGAS) were infected for 10 days with FGLenti-RT^{V148I} SIINFEKL. Spleen cells were stimulated for 6 hours with SIINFEKL peptide (10^{-13} to 10^{-8} M) prior to intracellular IFNγ staining and analysis by flow cytometry. Percentages of IFNγ^{+} activated (CD62^{lo}) CD8 T cells are shown.
(J-K) Wild type (C57Bl/6, n = 5) and *Samhd1*$^{-/-}$ mice (n = 5) were injected intramuscularly with 10μg OVA and 10μg cGAMP. (J) Quantification of H2-Kb-SIINFEKL tetramer^{+} CD8 T cells present in the spleen 10 days after challenge. Each dot represents an individual mouse. (K) Intracellular IFNγ staining of spleen cells from (J) after 6 hours of SIINFEKL peptide (10^{-13} to 10^{-8} M) re-stimulation. Percentages of IFNγ^{+} CD8 T cells are shown. The dashed line represents the fraction of IFNγ^{+} CD8 T cells responding to peptide in a mouse injected with PBS.
Data are representative of 2 (B-H) or 1 (I-K) independent experiment(s). Data in (B), (D-K) represent mean ± SD. *$p < 0.05$; unpaired t-test (B, D-H, J) and non-linear 4PL sigmoidal curve fitting (I, K).

Supplemental Tables

Table S1: oligonucleotides used for lentivirus plasmid cloning

Plasmid	Lentivirus	Oligonucleotide	Sequence: 5' to 3'
pNL4-3-deltaE-EGFP-RT^{V148I}	FGLenti-RT^{V148I}	Fwd-left-ApaI	ATTGCAGGGCCCCTAGGAAAAAGGGCT
		Rvs-left-V148I	CCATCCCTGTGGAAGGATATTGTACTGATATCT
		Fwd-right-V148I	AGATATCAGTACAATATCCTTCCACAGGGATGG
		Rvs-right-EcoRI	GTTGCAGAATTCTTATTATGGCTTCCACTCC
pNL4-3-deltaE-EGFP-RT^{V148I} SIINFEKL	FGLenti-RT^{V148I} SIINFEKL	Fwd-left-AatII	CCACCTGACGTCTAAGAAACCATTA
		Rvs-left-SIINFEKL	CAGCTTCTCGAAGTTGATGATGCTTGTGTCAGCTG CTGCTTGCTGTGCCTT
		Fwd-right-SIINFEKL	AGCATCATCAACTTCGAGAAGCTGGGAAACAACA GCCAGGTCAGCCAAAATTAC
		Rvs-right-ApaI	TCCTAGGGGCCCTGCAATTTTTGGCTA

Table S2: Plasmid combinations for lentivirus production

Lentivirus	Plasmids
FGLenti	pNL4-3-deltaE-EGFP and pMD2.G
FGLenti-RT^{V148I}	pNL4-3-deltaE-EGFP-RT^{V148I} and pMD2.G
FGLenti-RT^{V148I} SIINFEKL	pNL4-3-deltaE-EGFP-RT^{V148I} SIINFEKL and pMD2.G
SGLenti-RT^{V148I}	pRRLsin.EGFP, pCMVΔ8.2 RTV148I and pMD2.G
VLP(-Vpx)	pSIV4+ and pMD2.G
VLP(+Vpx)	pSIV3+ and pMD2.G

Table S3: TaqMan probes (Applied Biosystems)

**Gene**	**Assay Probe ID**
Ifi44	Mm00505670_m1
Ifit1	Mm00515153_m1
Ifit2	Mm00492606_m1
Oasl1	Mm00455081_m1
Isg15	Mm01705338_s1
Gapdh	Cat. no. 4352932E
EGFP	Mr04329676_mr
Tfrc	Cat. no. 4458366

Table S4: antibodies used for Western blot

**Antigen**	**Supplier**	**Host**	**Cat. number**	**Dilution**
HIV-1 p17	NIH AIDS Reagent Program	Rabbit polyclonal	4811	1:2,000
HIV-1 p24	Advanced Bioscience Laboratories	Mouse monoclonal	4313	1:5,000
Human SAMHD1	Abcam	Mouse monoclonal	Ab67820	1:1,000
Mouse SAMHD1	In house (Rehwinkel et al., 2013)	Rabbit polyclonal		1:100,000
Beta-actin	Sigma Aldrich	Mouse monoclonal (HRP-coupled)	A3854	1:100,000
ISG15	A. Pichlmair	Rabbit polyclonal		1:2,000

Table S5: antibodies used for flow cytometry

Cell surface marker	Supplier	Clone
Anti-human CD86	eBioscience	IT2.2
CD11c	eBioscience	N418
MHC-II	eBioscience	M5/114.15.2
CD80	eBioscience	16-10A1
CD86	eBioscience	GL1
SIINFEKL-H-2Kb	eBioscience	25-D1.16
CD62L	eBioscience	MEL-14
B220	eBioscience	RA3-6B2
CD40	Biolegend	3/23
IFNγ	BD Biosciences	XMG1.2

Supplemental Experimental Procedures

Plasmids and reagents

Nevirapine (#4666), raltegravir (#11680) and the pNL4-3-deltaE-EGFP plasmid (#11100; FGLenti) were obtained through the NIH AIDS Reagent Program, Division of AIDS, NIAID, NIH. pNL4-3-deltaE-EGFP was from Drs. Haili Zhang, Yan Zhou, and Robert Siliciano (Zhang et al., 2004). Plasmids for FGLenti-RT^{V148I} and FGLenti-RT^{V148I} SIINFEKL were cloned using pNL4-3-deltaE-EGFP as a template *via* overlap PCR using the oligo's listed in table S1. pMD2.G, pRRLsin.EGFP (SGLenti) and pCMVΔ8.2-RT^{V148I} have been described before (Rehwinkel et al., 2013). pSIV3+ and pSIV4+ are described in (Negre et al., 2000). IFNα A/D was purchased from Sigma Aldrich. SeV (Cat. No. VR-907) was purchased from ATCC.

Cells

BMMCs, BMDMs and human MDDCs were cultured in RPMI (Sigma Aldrich) supplemented with 10% heat inactivated FCS, 2 mM L-Gln, 100 U/ml penicillin, 0.1 mg/ml streptomycin and 50 μM 2-mercaptoethanol. For BMMCs and BMDMs bone marrow was cultured with 20 ng/ml GM-CSF (Peprotech) or 20% L929 supernatant. Medium was partially refreshed at day 2 and replaced entirely on day 3. Cultures contained on average 60-70% CD11c$^+$ MHC-II$^+$ cells or > 95% CD11b$^+$ F4/80$^+$ macrophages. For detection of the spontaneous IFN response, BMDMs were harvested at day 5 and cultured for an additional 7 days in RPMI medium supplemented with 20% L929 supernatant. Human MDDCs were differentiated for 5 days from CD14$^+$ monocytes with 40 ng/ml GM-CSF and 40 ng/ml IL-4. Monocytes were purified from total PBMCs using MACS separation columns and CD14 microbeads (Miltenyi). The cultures contained > 95% DC-SIGN$^+$ cells. TLA HEK293T cells (Open Biosystems) were grown in DMEM (Sigma Aldrich) supplemented with 10% heat inactivated FCS and 2 mM L-Gln. All cells were grown at 37°C and 5% CO_2.

Detection of IFN and CXCL10

Mouse IFNα was detected by ELISA. Anti-mouse IFNα antibody (clone F18, Hycult Biotech) and anti-mouse IFNα antibody (PBL Assay Science, 32100) were used as capturing and detection antibodies and IFNα (Hycult Biotech, HC1040b) as a standard. Alternatively, IFNα was measured by LumiKine IFNα ELISA (Invivogen). CXCL10 ELISA was from eBioscience. The HEK293-ISRE-luciferase based bioassay described in (Bridgeman et al., 2015) was used for the detection of human IFN. Human IFNα2 (R&D Systems) was used as a standard.

qPCR and RT-qPCR

Cells were lysed in RLT buffer, homogenized using a Qiaschredder (Qiagen) and purified using RNeasy columns with DNase I digestion (Qiagen). Tissues were homogenized with glass beads (Sigma Aldrich) in TRIzol (Thermo Fisher) on a FastPrep F120 instrument (Thermo Fisher). After chloroform phase-separation, RNA (aqueous phase) was further purified using RNeasy columns. cDNA synthesis was performed with SuperScript II reverse transcriptase (Thermo Fisher) and random hexamer primers (Ambion). For DNA extraction, cells were lysed in AL-buffer and incubated with proteinase K at 56°C for 10 min and purified using DNeasy blood and tissue columns (Qiagen). 15 ng of input material was amplified using TaqMan universal PCR or Taqman Genotyping Master Mix (for *Tfrc*) on a 7800 real-time PCR system (Applied Biosystems). Expression data were normalized to *Gapdh* (mRNA) or *Tfrc* (cDNA) and analyzed by the comparative C_t method. The TaqMan probes (Applied Biosystems) used in this study are listed in table S3.

Western blot and FACS

Cells and virus preparations were lysed in 25mM Tris.HCl pH 7.4, 150mM NaCl, 2mM EDTA, 1% Igepal CA-630 (Sigma Aldrich) and 5% glycerol and protease inhibitor cocktail (Cell Signalling). Protein lysates were cleared by centrifugation at 16,000g for 10 min. Antibodies used for Western blot are listed in table S4. Anti-rabbit or anti-mouse antibodies coupled to HRP were from GE Healthcare Life Science. Anti-mouse SAMHD1 was described in (Rehwinkel et al., 2013). Antiserum to HIV-1 p17 (4811) was obtained through the NIH AIDS Reagent Program, Division of AIDS, NIAID, NIH from Dr. Paul Spearman (Varthakavi et al., 1999). For FACS analysis, BMMCs were detached with Accutase (Sigma Aldrich). Blood was subjected to red blood lysis (ACK-buffer, Sigma Aldrich). Spleens were digested with 100 μg/ml collagenase type IV (Worthington) and 20 μg/ml DNase I (Roche). Cells were stained in FACS buffer (PBS, 2mM EDTA, 1%FCS). 1 μg/ml DAPI (Sigma Aldrich) or Live/Dead Fixable Violet (Molecular Probes) was used to exclude dead cells. Data were acquired on Beckmann Coulter CyAn

or BD Biosciences LSRFortessa flow cytometers. The antibodies used for FACS are listed in supplementary table 5. H-2K^b-SIINFEKL tetramer was a gift from V. Cerundolo.

Supplemental References

Negre, D., Mangeot, P.E., Duisit, G., Blanchard, S., Vidalain, P.O., Leissner, P., Winter, A.J., Rabourdin-Combe, C., Mehtali, M., Moullier, P., *et al.* (2000). Characterization of novel safe lentiviral vectors derived from simian immunodeficiency virus (SIVmac251) that efficiently transduce mature human dendritic cells. Gene therapy *7*, 1613-1623.

Varthakavi, V., Browning, P.J., and Spearman, P. (1999). Human immunodeficiency virus replication in a primary effusion lymphoma cell line stimulates lytic-phase replication of Kaposi's sarcoma-associated herpesvirus. Journal of virology *73*, 10329-10338.

Zhang, H., Zhou, Y., Alcock, C., Kiefer, T., Monie, D., Siliciano, J., Li, Q., Pham, P., Cofrancesco, J., Persaud, D., and Siliciano, R.F. (2004). Novel single-cell-level phenotypic assay for residual drug susceptibility and reduced replication capacity of drug-resistant human immunodeficiency virus type 1. Journal of virology *78*, 1718-1729.
